# Genetic Variability, Heritability, and Clustering Pattern Exploration of Bambara Groundnut (*Vigna subterranea* L. Verdc) Accessions for the Perfection of Yield and Yield-Related Traits

**DOI:** 10.1155/2020/2195797

**Published:** 2020-12-19

**Authors:** Md Mahmudul Hasan Khan, Mohd Y. Rafii, Shairul Izan Ramlee, Mashitah Jusoh, Al Mamun

**Affiliations:** ^1^Laboratory of Climate-Smart Food Crop Production, Institute of Tropical Agriculture and Food Security (ITAFoS), Universiti Putra Malaysia (UPM), 43400, UPM Serdang, Selangor, Malaysia; ^2^Department of Plant Breeding, Bangladesh Agricultural Research Institute (BARI), Gazipur 1701, Bangladesh; ^3^Department of Crop Science, Faculty of Agriculture, Universiti Putra Malaysia (UPM), 43400, UPM Serdang, Selangor, Malaysia; ^4^Breeding Divison, Bangladesh Jute Research Institute (BJRI), Dhaka 1207, Bangladesh

## Abstract

Bambara groundnut (*Vigna subterranea* L. Verdc.) is considered an emerging crop for the future and known as a crop for the new millennium. The core intention of this research work was to estimate the variation of landraces of Bambara groundnut considering their 14 qualitative and 27 numerical traits, to discover the best genotype fitted in Malaysia. The findings of the ANOVA observed a highly significant variation (*p* ≤ 0.01) for all the traits evaluated. There was a substantial variation (7.27 to 41.21%) coefficient value, and 14 out of the 27 numerical traits noted coefficient of variation (CV) ≥ 20%. Yield (kg/ha) disclosed positively strong to perfect high significant correlation (*r* = 0.75 to 1.00; *p* ≤ 0.001) with traits like fresh pod weight, dry pod weight, and dry seed weight. The topmost PCV and GCV values were estimated for biomass dry (41.09%) and fresh (40.53%) weight with high heritability (Hb) and genetic advance (GA) Hb = 95.19%, GA = 80.57% and Hb = 98.52%, GA = 82.86%, respectively. The topmost heritability was recorded for fresh pod weight (99.89%) followed by yield (99.75%) with genetic advance 67.95% and 62.03%, respectively. The traits with Hb ≥ 60% and *GA* ≥ 20% suggested the least influenced by the environment as well as governed by the additive genes and direct selection for improvement of such traits can be beneficial. To estimate the genetic variability among accessions, the valuation of variance components, coefficients of variation, heritability, and genetic advance were calculated. To authenticate the genetic inequality, an unweighted pair group produced with arithmetic mean (UPGMA) and principal component analysis was executed based on their measurable traits that could be a steadfast method for judging the degree of diversity. Based on the UPGMA cluster analysis, constructed five distinct clusters and 44 accessions from clusters II and IV consider an elite type of genotypes that produce more than one ton yield per hectare land with desirable traits. This study exposed an extensive disparity among the landraces and the evidence on genetic relatives will be imperative in using the existing germplasm for Bambara groundnut varietal improvement. Moreover, this finding will be beneficial for breeders to choose the desirable numerical traits of *V*. *subterranea* in their future breeding program.

## 1. Introduction

The Bambara groundnut (*Vigna subterranea* L. Verdc.; Syn: *Voandzeia subterranea* L. Thouars) is an underutilized grain legume that belongs to the family of Fabaceae and subfamily of Faboidea grown mostly in Africa [1]. Verdcourt [2] suggested the present binomial name *Vigna subterranea* (L.) Verdc and its chromosome number is 2*n* = 2*x* = 22 [3]. Bambara groundnut is a future emerging legume grown in Africa and Asia, is commonly referred to as a poor man's crop, mostly known as “Women's Crop” for family food security [1], and recently noticed as the crop for new millennium [4]. The Bambara groundnut is treated as the 3rd most important legume crop after groundnuts (*Arachis hypogaea* L) and cowpeas (*Vigna unguiculata* L. Walp.) in Africa [5] but due to its low rank, it is considered a snack or food supplement but not a lucrative cash crop [6]. The center of origin of Bambara groundnut is believed to be ‘Bambara,' a place name near Timbuktu in central Mali, West Africa [7], and suffix ‘groundnut' is because the process of its pods grows under soil, which is equal to peanut/groundnut; hence, its common name is ‘Bambara groundnut.' The crop has been vastly cultivated in tropical areas, now available in several parts of South America, Asia, and Oceania [8]. Effectively, it is cultivated for human utilization and has been noted as a fully balanced diet due to a high content of carbohydrate (63-65%), protein (18-20%), and oil (17-18%) in its seed [9, 10]. The biochemical investigation reported that it possesses essential amino acids (33.31%) and nonessential amino acids (66.69%) out of the total content [11, 12]. Bambara groundnut has great potential for incorporation into different foods of human where it possesses [11] crude protein (17.5 to 21.1%), crude fat (7.3 to 8.5%), total ash (4-5%), crude fiber (1.8-2.0%), CHO (53.0 to 60.8%), and moisture (7.5 to 12.3%). The mineral content of Bambara groundnut seeds that was accounted for (mg/100 g dry matter) the macro minerals are Ca (37-128), K (1545-2200), Mg (159-335), Na (16-25), P (313-563), and for the micro minerals (ppm) Cu (3.0-13.2), Fe (23.0-150) and Zn (13.9-77.0) reported by Amarteifio et al. [12]. Due to the high content of iron and protein with a significant level of lysine (10.3%), methionine seeds of Bambara were marked as uniformed food sources when compared to other food legume crops [13]. For its rich protein content, Bambara groundnut fulfills the daily demand of proteins for the low-income users and also has the potentiality to develop nutrition and food security and accelerate rural improvement and sustainable use of land where animal proteins are not freely available for service as a staple food for them [14]. In some areas of Nigeria [15], the young fresh seeds were boiled and eaten as a manner of the way like boiled peanut was also made a pudding known as Moi-Moi or Okpa or porridge bean. Bambara groundnut is used for making bread [16], and roasted seeds are eaten as confectionery [17], pounded and mixed with soup [18]. Habitually, the use of raw bean in a manner of chewing and swallowing prevent nausea [19], green leaves are eaten for antivomiting [9, 20], and its fodders are used to feed for animals [21]. Bambara groundnut was treated as a source of fully balanced food [22] and permitted to grow in drought with various agroecological aspects resulting to it becoming an important economic crop for developing countries [23]. Same as other legume crops, it can fix atmospheric nitrogen [24], mostly grown by the female [25] and able to produce a high yield with low input. In Swaziland, it is reported that about 98% of farmers regard Bambara groundnuts as a profitable crop [26] and most of the portion of total yield is used by themselves; the rest the amount (10-40%) was sold to the local market. Globally, the estimated annual production of Bambara groundnut was 160,378 tons in which 111,562 tons are produced in West Africa [27]; besides, Burkina Faso occupied the major portion in Africa with Nigeria leading its production at 100,000 metric tons per annum [28]. However, the existing landraces produce low yield (650 kg/ha) due to lack of following appropriate farming methods, diseases-insect infestation, and lack of improving genotype best adjusted to climate change [1, 29]. On the other hand, some researchers [30] have noted that improved Bambara groundnut genotypes can produce yield 3.0 t/ha to 4.5 t/ha when all factors related to yield are favourable in condition. The adequate knowledge of several genotypes and their evaluation is obligatory for germplasm selection as well as their enhancement approaches [31]. The global population growth rate increases by an estimation of 80 million per year and assume to reach 9.2 billion by 2050 [32]. So, based on the current situation use of potential genetic resources for plant breeding to boost up the production of this crop [33] which can provide as a supplement to meet up a certain defect in the consumption of major crops like rice, wheat and maize also enhance food security in developing countries. Baudoin and Mergeai [8] reported that the Bambara groundnut is an extreme autogamous crop with cleistogamous flowers [34]. Many researchers noted that the effective hybridization between two different lines of Bambara groundnut through traditional breeding has not yet been achieved [13, 35]. The traditional breeding technique for the enhancement of the Bambara groundnut is slow and problematic due to the long generation time and preponderant homozygous nature of the crop [36]. In addition to geotropic pod formation of the Bambara groundnut, it makes its trouble to artificial hybridization [37]. The breeding technique of Bambara groundnut is undefined, and there are no high-yielding cultivars available in Malaysia and local landraces of this crop are still grown. Henceforth, the readily accessible development approach is to apply the selection technique to the already existing variable accessions. The current demand is to discover modern high-yielding cultivars for certain growing regions [38]. Morphological characterization is the first footstep of the germplasm investigation to identify desirable traits of interest [39]. Despite the versatile advantages of Bambara groundnut cultivation, a few findings have experimented on this edible crop in Malaysia compared to the other legumes like sorghum, groundnut, and cowpea. But like other African countries, the Bambara groundnut is vastly grown in Nigeria as a legume crop [40]; besides the African continent, the secondary center of cultivation of Bambara groundnut is in the Asian region like Sri Lanka, Malaysia, Philippines, India, and Brazil [21]. Bambara groundnut can well adapt to the tropical area like Malaysia, where the cultivation of major crops (rice, wheat, maize, etc.) are increasingly challenging due to drought and unpredictable rainfall patterns [41]. This research work emphasize the morphological performance of 150 Bambara groundnut accessions with a view of exploring the variation that exists among the traits with an aim of selection for high yield and further help to the selection of elite genotypes in breeding and agricultural improvement programs. In the current background of global climate change, one of the best approaches to reduce the hereditary erosion of the Bambara groundnut is the germplasm collection and diversity analysis in different growing regions. It could assist to identify the ongoing cultivating landraces as well as to inaugurate the way of management and upgrading of varietal characters. Consequently, this research discovered that the morphological divergence exists in Bambara groundnut accessions in Malaysia. Currently, the available modern strategies are applying for the selection of ongoing cultivated species of Bambara groundnut. After all, the trait improvements that can be made through direct selection are estimated by the availability of heritable variation. Furthermore, heritability influences the magnitude of the selection procedure which would be a powerful tool to improve a certain trait and predict the genetic gain from selection also estimating the comparative effect of genes [42]. Therefore, the pinpoint of this research was to discover the genetic divergence in different qualitative and numerical traits of Bambara groundnut with a certain goal (like high yield) of determination of variance component, heritability, genetic advance, and clustering, based on the selection intensity of the accessions with potentially high-yielding criteria.

## 2. Materials and Methods

### 2.1. Experiment Location

This research has been conducted from September 2018 to February 2019 at field-15 at University Putra Malaysia. The research work has experimented under the Institute of Tropical Agriculture and Food Security (ITAFoS), University Agricultural Research Park, University Putra Malaysia (UPM), Malaysia. Based on the Global Positioning System (GPS), the research location was 2°58′54.0′′N latitude and 101°42′53.8′′E longitude. The seeds of accessions were sown in open field conditions during the 2018-2019 cropping season. During the planting season, the mean climatic situation is stated in Table 1. The soil pH is 6.6 to 7.5 with sandy loam to clay loam type (Dept. of land management, UPM).

### 2.2. Genetic Materials

One hundred fifty accessions of Bambara groundnut were selected for this current research work, all representing the African accessions collected from the local market of Nigeria. The list of Bambara groundnut accession used in this research was displayed in Table 2. To estimate the genetic divergence using the morphophysiological traits, five randomly plants were taken for data investigation among the accessions evaluated [43].

### 2.3. Experimental Design

The experiment was conducted in a randomized complete block design (RCBD) with three replications. The experimental plot comprised of single rows measuring 2.10 m × 0.80 m. The distance between the plant to the plant was 30 cm and row to row distance was 1 m and the distance between replication was 2.0 m according to [43]. During the growing season, the recommended intercultural practices like land preparation, land clearing, weeding, irrigation, and fertilizer were approved. Hand sowing was done into a raised seedbed, with two seeds in a single hole in 3 cm depth, and the seedlings were uprooted to keep in one plant per hole after 2 weeks of sowing when seedlings are completely stable with soil. The recommended fertilizer rates (100% N = 45 kg N/ha, 100% P = 54 kg P_2_O_5_/ha, and 100% K = 45 kg K_2_O/ha). The total portion of phosphorus (100% P) and potassium (100% K) was applied during land preparation; hence, 70% N was applied at 5 weeks after sowing [44].

### 2.4. Parameters Measured for Data Analysis

Both qualitative and quantitative data were taken following the Bambara groundnut descriptors [45]. Twenty-seven measurable characters (Table 3) and fourteen qualitative characters (Table 4) were considered during the morphological characterization. The measurable traits were divided into the following three categories: (1) phenological traits, (2) growth and vegetative traits, and (3) yield traits to easy interpretation. All parameters were visually recorded at different growth stages of five plants in the field and after harvest in the lab as per the following description and descriptors states [45].

### 2.5. Statistical Analysis

The SAS (statistical analysis software) version 9.3 was followed to test the significant differences using the analysis of variance (ANOVA) procedure at the level of LSD (*p* ≤ 0.05) and to compare among the means of significant traits. The result of this research was expressed as mean, the square of mean, genetic parameter, and lastly, the Pearson correlation was measured to find out the intercorrelation ships among the traits. The correlations between the quantitative variables were determined using the Pearson [46] correlation coefficient formula. A report from [37] noted that correlation is an appropriate guide, particularly for plant breeders who may want to associate a set of traits in their selection programs. Typically, the correlation studies among numerical traits are of great value to plant breeders in selecting elite traits.

## 3. Genetic Parameter Analysis

### 3.1. Estimation of Covariance, Broad-Sense Heritability, and Genetic Advance

(1) Analysis of variance (ANOVA) estimation: this analysis was done to discover the uniqueness among the accessions and estimate the effects of environment and genes on several traits
(2) The genotypic and phenotypic variation was calculated as per following the formula given by [47]:
  (1) $$\begin{array}{c} \;\sigma_{g}^{2}=\frac{\text{MSG}–\text{MSE}}{r},\\ \sigma_{p}^{2}=\sigma_{g}^{2}+\text{MSE}, \end{array}$$
  where *σ*_*g*_^2^ is the genotypic variance, *σ*_*p*_^2^ is the phenotypic variance, MSG is the genotypic mean square, MSE is the error mean square, and *r* is the replication number
(3) The coefficient variation of phenotypic (PCV) and genotypic (GCV) were estimated as per formula given by [48]:
  (a) $\text{PCV}=\left(\sqrt{\sigma_{\text{p}}^{2}}/\bar{X}\right)\times 100$
  (b) $\text{GCV}=\left(\sqrt{\sigma_{\text{g}}^{2}}/\bar{X}\right)\times 100$
  (c) RD = (PCV − GCV/PCV) × 100
  where PCV is the phenotypic coefficient of variation, GCV is the genotypic coefficient of variation, $\bar{X}$ is the grand average of the traits, *σ*_*p*_^2^ is the phenotypic variance, *σ*_*g*_^2^ is the genotypic variance, and RD is the relative difference between PCV and GCV.
The estimated values of PCV and GCV were categorized by [48, 49] like as between 0%-10% for low, 10%-20% for intermediate, and greater than (≥20%) for high.
(4) Broad sense heritability (*h*_*b*_^2^): it refers to the proportion of genotypic variance (*σ*_*g*_^2^) with the phenotypic variance (*σ*_*p*_^2^) multiplied by a hundred. For estimation of (*h*_*b*_^2^), the formula given by [50] was followed:
  (2) $$\begin{array}{c} h_{b}^{2}\left(\%\right)=\frac{\sigma_{g}^{2}}{\;\sigma_{p}^{2}}\times 100 \end{array}$$
  where *σ*_*g*_^2^ = Genotypic variance and  *σ*_*p*_^2^= Phenotypic variance. In accordance with [51, 52], the heritability grade was ordered between 0% and 30% for low, 30% and 60% for intermediate, and greater than 60% as high.
(5) Genetic advance (GA) (as a percentage of mean) was calculated with a 5% selection intensity (*K*) following the method of [51]. Genetic advance is categorized as between 0% and 10% for low, 10% and 20% for intermediate, and more (>20%) than for high, following the formula given by [53]:
  (3) $$\begin{array}{c} \text{GA}\left(\%\right)=K\times \frac{\sqrt{\sigma_{p}^{2}}}{\bar{X}}\times h_{b}^{2}\times 100,, \end{array}$$
  where *K* is the constant that indicates the intensity of selection. According to Adewale et al. [54], the rate is 2.06 at the point when the *K* is at 5%. $\sqrt{\sigma_{p}^{2}}$ is the standard deviation of phenotype, *h*_*b*_^2^ is the broad sense heritability, and $\bar{X}$ is the grand mean values of traits.
(6) Genetic gain (%): estimated as genetic advance (GA) × 100; it is also categorized [51] as between 0 and 10% for low, 10 and 20% for intermediate, and ≥20% for high genetic advance

Further, to determine the expected gain from selection, estimation of heritability simultaneously with the values of the genetic advance can be an effective tool of crop enhancement program. Valuation of this component is an extensively fundamental step that must be taken into consideration before commencing any breeding program. In our current research, the enhancement of Bambara groundnut yield preference of the selection method was taken based on the magnitude of variation that exists in the gene pool of this crop, measurement of variance component, heritability, and genetic advance.

### 3.2. Multivariate Analysis

To determine the genetic or hereditary divergence, cluster analysis was used for 27 numerical traits that were considered in this study. Based on the Euclidian distance method, data was analyzed for investigation of genetic diversity. In addition to this, based on the unweighted pair group method using arithmetic average (UPGMA) and following the algorithm and sequential, agglomerative, hierarchic, and nonoverlapping (SAHN) method, the genetic interrelationship among the Bambara groundnut was estimated using the SAS version 9.3 software. Using similar software, the principal component analysis (PCA) was done with subsequent correlation coefficients applied to construct a dendrogram to observe the groupings and relatedness among the accessions of the Bambara groundnut. The NTSYS version 2.1 (Numerical Taxonomy Multivariate Analysis System) and Exeter Software (Setauket, NY, USA software) [55] were used to produce two-dimensional (2D) plots for principal component analysis (PCA). Modena's [56] stopping rule was followed to select the number of clusters with Milligan and Cooper's [57] correction.

### 3.3. Shannon Diversity Index (*H*) and Evenness (*E*)

Diversity indexes are statistics regarded to summarize the diversity of a population in which each member belongs to a unique group. Shannon's diversity index (*H*) is another index that is generally used to categorize the species diversity in a certain community. Shannon's diversity index is an account for both richness and evenness present in the species also used for a wide diversity of fields. It is also known as phylogenetic indices or phylogenetic metrics, which is a numerical estimation that indicates how many types (such as species) of variation are present in a community and simultaneously can consider the phylogenetic relations among the individuals. However, the Shannon evenness index is a synonym for the Shannon equitability index and was calculated [58, 59] using the formula as follows:

(4) $$\begin{array}{c} H=-\overset{s}{\underset{i=1}{\sum }}\left[\left(\text{pi}\right)*\ln\left(\text{pi}\right)\right],\\ E=\frac{H}{H_{\max}}\left[H_{\max}=\ln\left(N\right)\right], \end{array}$$

where *H* is Shannon's diversity index, *H*_max_ is the maximum diversity possible, ln is the natural logarithm of a number, pi is the proportion of the population made up of species ‘*i*', *S* is the number of species or species richness in a sample, *N* is the number of total samples, and *E* = equitability = evenness = *H*/*H*_max_.

## 4. Result

### 4.1. Qualitative Diversity

**Figure 1:**
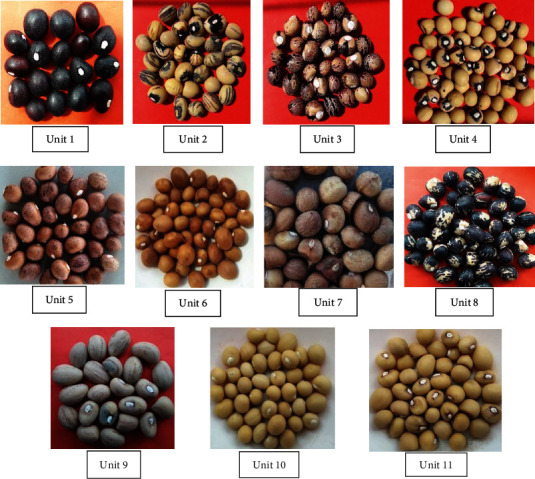
Different morphotypes of Bambara groundnut landraces. Unit 1: 14 accessions; Unit 2: 16 accessions; Unit 3: 13 accessions; Unit 4: 13 accessions; Unit 5: 13 accessions; Unit 6: 13 accessions; Unit 7: 13 accessions; Unit 8: 13 accessions; Unit 9: 16 accessions; Unit 10: 13 accessions; Unit 11: 13 accessions.

**Figure 2:**
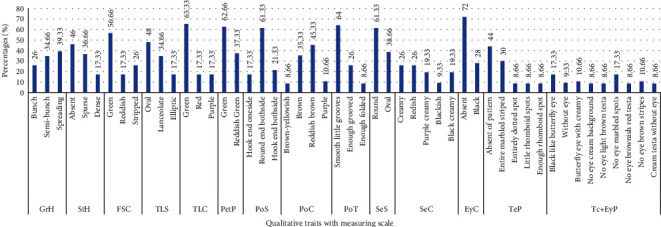
Summary of the frequency distribution of fourteen qualitative traits of Bambara groundnut accessions. GrH: habits of growth; StH: stem hairiness; FSC: first stem color; TLS: terminal leaflet shape; PetP: petiole pigmentation; PoS: pod shape; PoC: pod color; PoT: pod texture; SeS: seed shape; SeC: seed color (SeC); EyC: eye color; TeP: testa pattern; Tc+EyP: testa color with an eye pattern round hilum.

**Figure 3:**
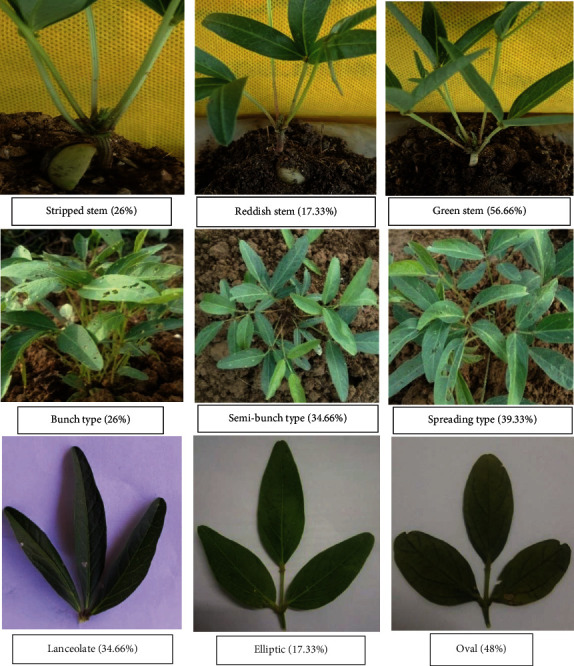
Some qualitative variables of Bambara groundnut.

The one hundred fifty landraces of Bambara groundnuts were taken from the local market of Nigeria in different growing regions. The accessions were grouped in 11 units (Table 5) by morphotypes (Figure 1), indicative therefore of the existence of many duplicates in the landraces based on the seed morphological description. The morphological characterization (Figures 2 and 3) summarized the frequency of distribution of some qualitative variables studied in this research. After two weeks, 56.66% of the accessions had greenish stems, 26% had stripped stems, and 17.33% were reddish stems. The terminal leaflets had three different colors: 65.33% accession had a greenish leaflet while the purple and red accessions were both 17.33%. 34.66% of the total accessions had terminal leaflets shaped like lanceolate whereas 48% had oval and 17.33% had elliptic in shape. Among the 150 characterized accessions, three growth habits were found (Figures 2 and 3): bunch-type accessions (26%), semibunch-type accessions (34.66%), and the spreading type (39.33%). Among the landraces, 36.66% had sparse hair on their stems and 17.33% had dense hair while 46% did not have any hair on their stems. Most of the landraces had reddish-brown (45.33%) and brown (35.33%) color pods; some had yellowish-brown (8.66%) and purple (10.66%) color pods. In this current research the seed's color and texture also taken into consideration and showed significant variation. Maximum accessions were found round (61.33%) shaped and few were oval (38.66%). Seed color had cream and red each of 26%, black-cream and cream-purple each of 19.33%, only 9.33% had black color. 28% of landraces had black eye color and 72% had no eye color. There was no testa pattern (44%) but entire line striped marbled (30%), enough rhomboid spots on both sides (8.66%) of the hilum, entirely dotted spot (8.66%), while little rhomboid spots on both side (8.66%) (Figure 2). Concerning the pod texture, most of the accessions (64%) had smooth little grooves while 26% had enough grooved and only 8.66% had enough folded. The existence of a significant morphological variation was detected for all the qualitative traits such as stem hairiness, terminal leaflet shape, growth habits, pods, and seed color.

### 4.2. Quantitative Traits

#### 4.2.1. Morphological Diversity

Most of the plant breeders treated yield and other yield contributing traits as high influential parameters for crop improvement. Generally, the traits which are associated directly and indirectly with yield are pod and seed size; shape; quality; plant height; branch number; the total number of pods, plant, and pod biomass weight; resistance to diseases and insect-pest infestation; and biotic and abiotic stresses that also have a significantly remarkable importance in Bambara groundnut breeding programs. However, in this current research, a total number of 27 quantifiable traits of 150 Bambara groundnut accessions were analyzed for the selection of best genotypes with high-yielding capacity. Significant variation, mean, standard error of the mean (SEm), standard deviation (St. Dev), and coefficient of variation (CV%) revealed by analysis of variance among the investigated 27 quantitative traits were displayed in Table 6. The analysis of variance (ANOVA) of 150 landrace's quantitative traits revealed highly significant (*p* ≤ 0.01) for all the traits studied among the accessions. For replication, all the variables showed a highly significant difference (*p* ≤ 0.01) except the variables no. of branch per plant, fresh biomass weight per plant, immature pod per plant, and shelling percentages observed no significant differences. The minimum and maximum values across overall plants were shown in Table 6, and the observed coefficient of variation (CV%) values ranged from 7.27% (days to maturity) to 41.21% (biomass dry weight per plant). The average days to maturity were found to be 130.89 ± 0.45 days which is statistically significant (*p* ≤ 0.01). The average no. of branches per plant with the standard error was observed 34.56 ± 0.33, for total no. of pod per plant 60.68 ± 0.48, for hundred seed weight 270.19 ± 1.56 and yield 1049.19 ± 14.93 kg/ha. The highest value of standard deviation (SD) was found for the trait yield kg/ha (SD = 316.64) with standard error (SEm: ±14.93) while the lowest was for internode length (SD = 0.70; SEm: ±0.03) (Table 6). We found significant differences in coefficient (CV ≥ 20%) for 14 traits out of the 27 numerical variables studied in this research. Days to 50% flowering varied from 25 to 52 days after sowing (DAS). We noticed that 70.66% of the accessions gave flowers before 40 DAS while 29.33% of the accession produced before 50 DAS. Most of the landraces (90%) had a life cycle of more than 120 days, and it was only one accession S1G141(0.66%) that took an average 160 days to maturity. Hundred seed weight varied between 196.21 g and 364.09 g whereas average highest hundred seed weight was calculated for the accessions S1G143 (329.58 g) and the lowest was S1G13 (203.72 g). As for the yield, ranged from 370.38 to 1679 kg/ha across the plant whereas topmost mean yield was recorded for the accession S1G92 (1635.29 kg/ha) while lowest was S1G28 (380.48 kg/ha). Lowest least significant difference (LSD = 0.05) was noticed for the trait internode length 0.36 while the highest was 35.28 for the trait no. of leaves per plant (Table 6).

#### 4.2.2. Performance of the Several Morphotype's Elite Accessions

**Figure 4:**
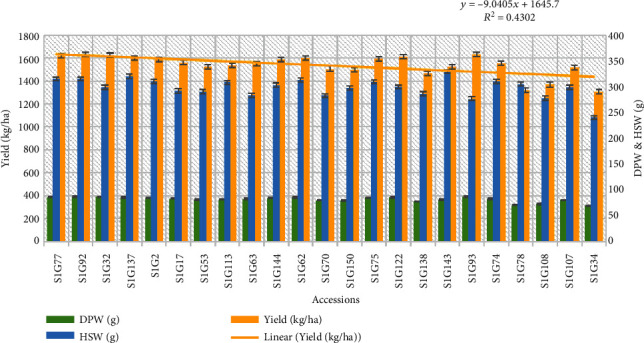
Graphical presentation dry pod weight (DPW) and hundred seed weight (HSW) with yield (kg/ha) for selected elite accessions of Bambara groundnut.

Twenty-three accessions were notified as elite stranded among the whole morphotypes based on a higher yield performance and other yield contributed quantitative traits (Table 7), and the at-a-glance relationship of dry pod weight (g) and a hundred seed weight (g) with yield (kg/ha) is displayed in Figure 4. All the 23 elite accessions gave the best field yield of more than one ton per hectare. The accessions S1G92 (1635.29 kg/ha) produced maximum yield followed by S1G93 (1632.87 kg/ha) and S1G32 (1626.41 kg/ha) among the accessions were studied. In addition to the high yield, the accession S1G108 (1369.01 kg/ha) was identified with a short life cycle of 106 days after sowing (Table 7).

### 4.3. Analysis of Correlation (Association) Matrix

The phenotypic association among the 27 numerical traits of one hundred fifty Bambara groundnut accessions is given in Table 8. No significant association was found for days to emergence with yield. Fifty percent flowering days had negative and weak (0.0 ≤ *r* < 0.25) significant correlation with yield (*r* = −0.22; *p* ≤ 0.001). Days to maturity had positive and intermediate (0.25 ≤ *r* < 0.75) highly significant association with yield (*r* = 0.29; *p* ≤ 0.001). Plant height had positive and weak (0.0 ≤ *r* < 0.25) highly significant relation with yield (*r* = 0.24; *p* ≤ 0.001) but intermediate correlation with seed length (*r* = 0.35; *p* ≤ 0.001). Significantly positive and intermediate association (0.25 ≤ *r* < 0.75) was found between biomass fresh weight (*r* = 0.37; *p* ≤ 0.001), biomass dry weight (*r* = 0.38; *p* ≤ 0.001), seed length (*r* = 0.37; *p* ≤ 0.001), and seed width (*r* = 0.34; *p* ≤ 0.001) with yield kg/ha. Correlation values for total no. of pod had positive and moderate (0.25 ≤ *r* < 0.75) highly significant association with yield (*r* = 0.68; *p* ≤ 0.001) while strong (0.75 ≤ *r* < 1.00) and significant (*r* = 0.93; *p* ≤ 0.001) relation was found with number of mature pod, dry seed weight (*r* = 0.94; *p* ≤ 0.001), and hundred seed weight (*r* = 0.88; *p* ≤ 0.001). Fresh pod weight detected positive and strong (0.75 ≤ *r* < 1.00) highly significant correlation with yield (*r* = 0.99; *p* ≤ 0.001) along with dry pod weight (*r* = 0.99; *p* ≤ 0.001) whereas dry pod weight showed positive perfect correlation (*r* = 1.00) with yield. The positive and intermediate (0.25 ≤ *r* < 0.75) highly significant association with yield was noted for hundred seed weight (*r* = 0.67; *p* ≤ 0.001) per plant (Table 8).

### 4.4. Genetic Parameter Analysis

#### 4.4.1. Variance and Covariance, Heritability in a Broad Sense, Relative Differences, and Genetic Advances

The investigation of covariance (genotypic and phenotypic), the genotypic coefficient of variation (GCV) and phenotypic coefficient of variation (PCV), relative differences (RD), broad-sense heritability, and genetic (GA) advance (as a percentage of mean) were displayed in Table 9. Briefly, the result exhibited that genotypic variance (*σ*_*g*_^2^) varied from internode length (0.25) to seed yield (100025). Unvaryingly, for the trait internode length, the least phenotypic variance ( *σ*_*p*_^2^), the value was 0.30 whereas for seed yield kg/ha was reported the topmost (100273.24) value. Seemingly, in the case of all traits, the phenotypic variance ( *σ*_*p*_^2^) is greater than genotypic variance (*σ*_*g*_^2^). Topmost PCV and GCV values were computed for biomass dry weight per plant and biomass fresh weight with a value of 41.09% and 40.53%, respectively. The lowest estimated values for GCV had 6.74% for shelling percent, whereas the lowest PCV values were found 7.26% for the trait days to maturity. The estimated both GCV and PCV values were found more than 20% for the traits such as number of stem per plant (PCV 23.51% and GCV 22.08%), biomass fresh (PCV 40.83% and GCV 40.53%) and dry (PCV 41.09% and GCV 40.09%) weight, fresh (PCV 33.02% and GCV 33%) and dry (PCV 30.18% and GCV 30.14%) pod weight, dry seed weight (PCV 26.31% and GCV 25.68%), and yield (PCV 30.19% and GCV 30.15%) kg/ha which indicate high variability among these traits and for the improvement of accession further selection could be done since the variation of these traits is due to the effect of additive genes. Rest of the traits showed GCV and PCV ≤ 20% although the traits days to maturity (PCV 7.26% and GCV 6.82%) and Shelling percent (PCV 8.47% and GCV 6.74%) showed below 10% coefficient of variation which indicates the limited scope of selection based on respected traits due to the effect of environment on their phenotypic expression.

*(1) Relative Difference (RD)*. The relative difference (RD) is referred to as an estimation of the ratio of GCV in association with the respective PCV and the estimated RD values varied from 0.05% (fresh pods weight) to 38.67% for no. of branch per plant (Table 9). The traits like no. of branch per plant (38.67%), no. of node per stem (22.55%), no. of immature pod per plant (27.39%), and pod width (32.93%) discovered a more difference in between their PCV and GCV values compared to other existing traits that indicated these traits had wider genetic variability due to environmental effect and not better feedback to direct selection for the improvement of traits. Oppositely, the traits like day to 50% flowering (3.03%), maturity date (6.08%), biomass fresh (0.74%) and dry (2.43%) weight, fresh (0.05%) and dry (0.12%) pod weight, dry seed weight (2.39%), harvest index (2.55%), and yield kg/ha (0.12%) had lower values of relative difference. Based on this result, it was noticed that the variation present among the traits because of a gene which has a better response to direct selection.

*(2) Heritability in a Broad Sense (hb2)*. Heritability refers to the ratio of the total variation of phenotypic traits in each population between the individuals due to genetic variation. Typically, the estimated values of heritability in the broad sense were high (*h*_*b*_^2^ > 30) for almost of the all traits evaluated (Table 9). The range of heritability broad sense for the estimated traits was varied from 37.67% (no. of branches per plant) to 99.89% (fresh pod weight). Generally, moderate (30% ≤ *h*_*b*_^2^ ≤ 60%) heritability values were marked for the traits like branch number per plant (37.61%), no. of immature pods per plant (52.73%), and pod width (44.98%) whereas the rest of the traits expressed high (h_b_^2^ ≥ 60%) heritability values, which indicate that the degree of heritability is less affected by the environment.

*(3) Genetic Advance (GA)*. Genetic advance (as percentage mean) was ranged between the lowest (11.06%) for shelling percent and biomass fresh weight (82.86%) (Table 9). Genetic advance for five traits such as days to maturity (13.19%), no. of branches per plant (16%), pod width per plant (14.34%), hundred seed weight (17.99%), and shelling percent (11.06%) showed intermediate genetic advance (10% ≤ GA ≤ 20%) whereas rest of the traits detected high (GA ≥ 20%) genetic advance values concurrently with high values of heritability. Superior GCV, alongside high heritability as well as high genetic advance, provides a superior indication of selection than the consideration of individual genetic matrix or measuring unit. The advancement of agronomic variables over selection was carried out based on the degree of the genetic variation that exists among the population. Besides this, it also depends on the strength of the transportability of certain traits which is a scale of estimation of heritability.

### 4.5. Cluster Analysis

**Figure 5:**
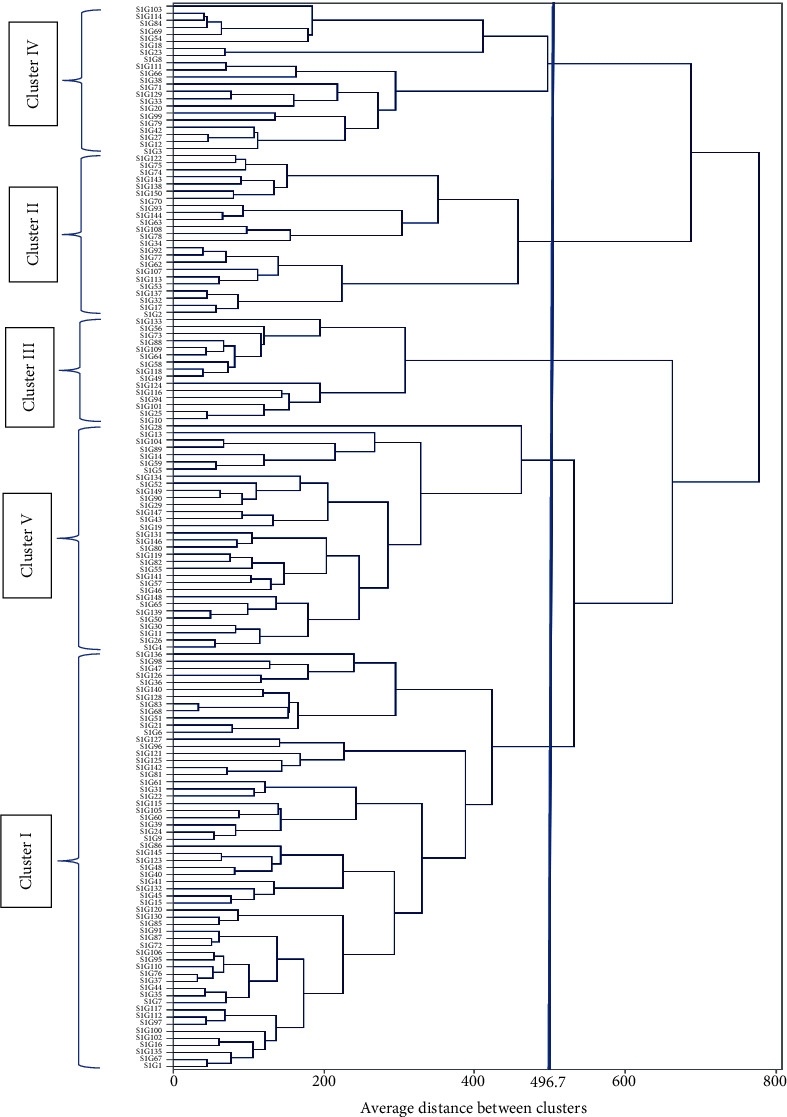
Cluster analysis revealed as dendrogram for 150 Bambara groundnut accessions based on the UPGMA method of SAHN clustering.

**Figure 6:**
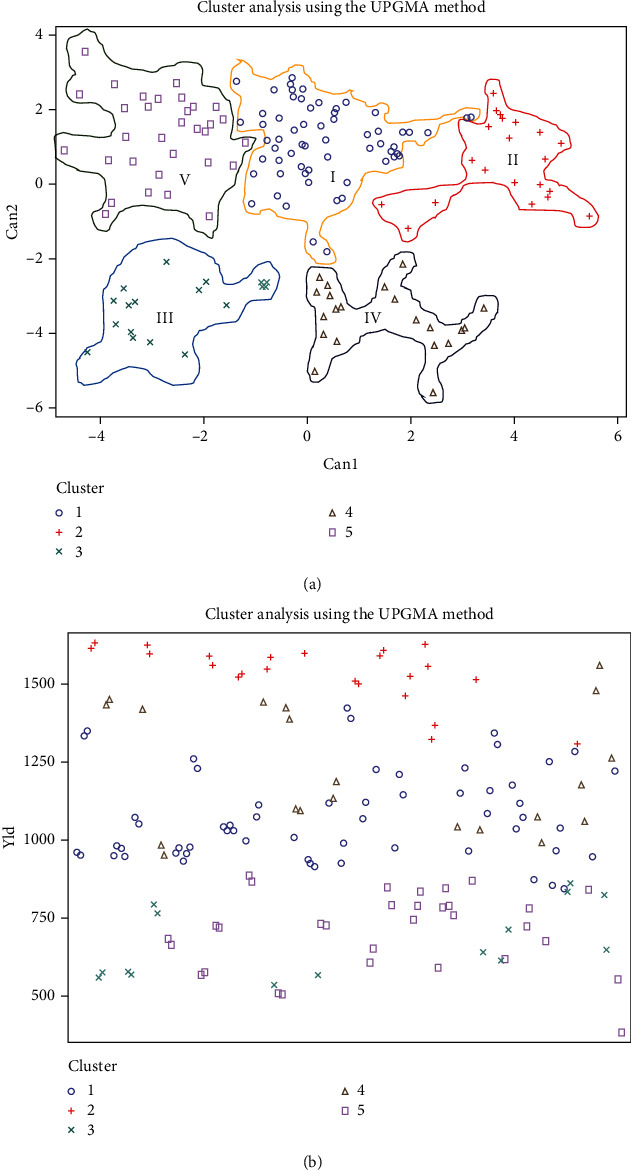
Graphical representation of different cluster (a) and relationship between genotype and yield (b) among the cluster revealed by UPGMA method cluster analysis.

For parental selection, genetic distinction analysis is one of the norms, in which the degree of divergence among obtainable landraces is demonstrated. Clustering provides a very strong and strict clue on the degree, and the nature of genetic divergence is notable for the selection of expected genotype. In this study, the homogenized data was used to calculate the Euclidean distances among the 150 Bambara groundnut accessions and the cluster analysis presented as a dendrogram (Figure 5) using UPGMA (average linkage cluster analysis) revealed numerous clusters depicting associations among these collected accessions. To categorize against the relations in the accessions, accessions were clustered into five major clusters (Figure 5) based on their twenty-seven measurable traits at the dissimilarity of 496.7. In the dendrogram, there was a cut off at the point of 496.7 for picking cluster number and ease of interpretation using Mojena's stopping rules. Cluster I recorded the highest number (39.33%) accessions with an average yield of 20.67% while maximum average yield was recorded for cluster II (29.45%) which consists of 23 accessions with best agronomic traits (Table 10) followed by cluster V (21% accessions) but lower yield of 13.56%. Cluster IV consists of 14% of the accessions with 2^nd^ average maximum yield (23.5%) while cluster III was constructed by only 10% of the total accessions with lowermost average yield (12.48%). Additionally, we recorded 46.51%, 16.90%, and 2.67% higher (+) mean yield compared to average grand mean yield (1049.19 kg/ha) for cluster II, cluster IV, and cluster I, respectively, while cluster III (36.11%) and cluster V (32.53%) gave lower (-) yield. So, cluster II (23) and cluster IV (21) had accessions adjacent to each other on the aspect of yield traits (Figures 6(a) and 6(b)). For this crop improvement, 44 accessions from cluster II and IV associated with large seed size and high yielding potential were marked as potential accessions.

### 4.6. Estimation of Principal Component Analysis

**Figure 7:**
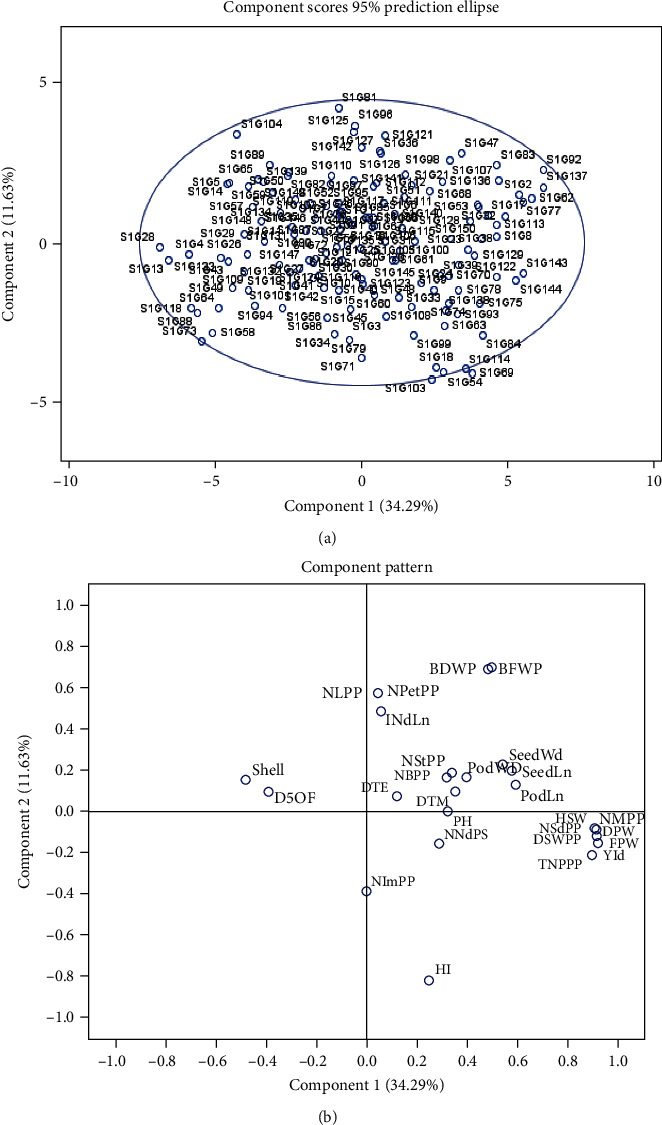
Graphically showing the principal component plot and their proportion of variance for 27 morphological traits.

**Figure 8:**
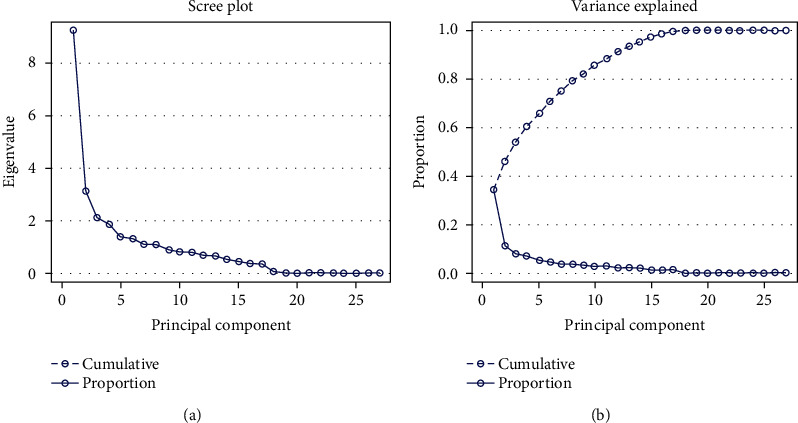
Graphical representation of position of 150 different Bambara accessions (a) and 27 morphological traits (b) revealed by PCA.

**Figure 9:**
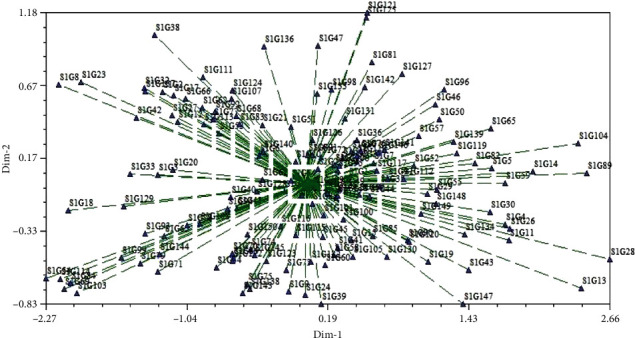
Two-dimensional (2D) graph showing the relationship among Bambara groundnut accessions using PCA revealed by NTYSIS.

Principal component analysis (PCA) has been vastly used in crop research for sorting the traits and grouping of accessions. In the current research first, eight principal components (PC) had accounted for 78.99% of the cumulative variation (Table 11 and Figure 7). The 1^st^ PC gained and recorded for the topmost proportion of the variance in the set of all PCs and rest for gradually smaller and smaller amounts of variation. However, the percent of variation for PC1 and PC2 was 34.29% and 11.63%, respectively, while the 9^th^ PC accounted for 3.28% of the variation. The graphical illustration of 150 accessions (a) and 27 morphological traits (b) exposed by PCA was shown in Figure 8. It is intended from Figure 8(b) 8-9 traits (total no. of pods, no. of mature pods, fresh pods weight, dry pod weight, no. of seed/plant, seed length, dry seed weight, hundred seed weight, and yield) and traits (petiole and leaves number, biomass fresh and dry weight, and seed length and width) had a positive correlation with PC1 and PC2, respectively. The traits contributing to PC1 and PC2 are showing the topmost variability with a high coefficient of variation as also exposed in an analysis of variance. The factor loading of several traits is displayed in Table 11 which were revealed by using PCA. The PC1 allowed loading of traits like total no. of pods, no. of mature pods, fresh and dry pods weight, no. of seed/plant, dry seed weight, hundred seed weight, and field yield indicating the significant for the respective principal components simultaneously, for PC2 (petiole and leaves number, biomass fresh and dry weight), for PC3 (seed length and width, harvest index, andplant height), for PC4 (days to emergence, stem number, pod width, and shelling %), PC5 (Ddays to 50% flowering, maturity date, seed length, and width), for PC6 (days to 50% flowering and maturity date), for PC7 (stems and branch number), for PC8 (plant height and internode length), and for PC9 (no. of nodes per stem and branch and stem number), showed significance to the respective PCs. The relationship between eigenvalues and principal component and their proportion of variation are shown in Figure 7. By considering these 8-9 PCs, it was exposed that these PCs regulate the total variation for all yield contributing traits. Furthermore, the two-dimensional graphical elucidation (Figure 9) demonstrated that most of the accessions were dispersed at low distances whereas the few were dispersed at high distances as reflected by an eigenvector (Table 11). The outermost accession from the centroid was S1G24, S1G13, S1G89, S1G54, S1G121, S1G125, S1G39, S1G147, and S1G4 whereas other accessions were near to the centroid.

### 4.7. Estimation of Shannon–Weaver Diversity (*H*′ Index)

The Shannon–Weaver diversity index was used to assess the phenotypic diversity for each trait. The estimation of the Shannon–Weaver diversity index (*H*) and evenness (*E*_*H*_) for the twenty-seven traits is shown in Table 11 using the formula [60]. The estimated Shannon–Weaver diversity index ranged from 4.93 to 5.01 among the traits evaluated. The equitability or evenness was found varied from 0.98 to 1.00. Among the traits, topmost (*H* = 5.01) diversity was estimated for maturity date and shelling percent followed by the traits like fifty percent flowering date, nodes number per stem, internode length, pod width, seed length, and seed width which (*H* = 5.00) indicated that maximum diversity was present among these traits while the lowermost diversity (*H* = 4.93) was noted for the trait biomass fresh and dry weight per plant. Similarly, maximum (*E*_*H*_ = 1.00) values of evenness were marked for almost all the traits whereas minimum (*E*_*H*_ = 0.98) was noted for biomass fresh and dry weight per plant.

## 5. Discussions

### 5.1. Qualitative Diversity

Effective selection is done when significant hereditary variation present with high magnitude among the accessions has been reported by Hahn [61] and Adebisi et al. [62]. Several researchers such as Mohammed [63], Sinise and Massawe [64], and Abu and Buah [65] noted a significant level of dissimilarity in numerical traits in Bambara groundnut. An observation among Bambara groundnut genotypes with these 3 types of growth habits was noticed by Ntundu et al. [1] in Tanzania and [30] in Cameroon which supported our research findings on qualitative traits. In the study of 52 landraces of Bambara groundnut by Gbaguidi et al. [66], it was concluded that the significant variation is present among all the qualitative traits, taken into consideration in his study. There was a similar observation by Ntundu et al. [1] that the farmers prefer bunch-type landraces compared to other reasons of it provide more advantages to the farmer especially during harvesting period where the roots and stems were unearthed. Vegetative growth of the Bambara groundnut is varied; our research result was equal with those of [67] who grouped Bambara groundnut into three categories namely, (bunche type, semibunch type, and spreading type) based on its vegetative growth. Similar types of growth habits were observed among the germplasms that were evaluated in this research work. For the Bambara groundnut crop, the qualitative trait growth habit is highly significant to the different cropping patterns [68]. The other qualitative traits which showed low variation did not display a rational distinct identity among the Bambara accessions studied.

### 5.2. Quantitative Traits

The findings of this work displayed that there was a vast genetic variation that exists among the Bambara groundnut accession estimated for twenty-seven numerical traits listed. The similar descriptive statistics analysis (averages, range, coefficient of variation, and standard deviation) confirmed the genetic diversity of the *Vigna subterranea* (L.) Verdc [1] and the cowpea (*Vigna unguiculata* L) [69]. Our research revealed that a high variation coefficient for traits like emergence time, biomass fresh and dry weight, branches number, fresh and dry pod weight, dry seed weight,and yield kg/ha. In the African continent, many researchers conducted intensive researches on Bambara groundnut using several morphological traits and their findings were supported by research findings such as genetic diversity and population structure of the Bambara groundnut improvement program [70]. Relevant observation of high variation coefficients was confirmed by Goli et al. [60] and indicates the existence of a massive heterogeneity among the landraces recorded in Cote d'Ivoire. In the variability test between local and exotic Bambara groundnut [71] in Botswana, Bambara groundnut showed a significant morphological variation reported by Bonny and Dje [72] and Touré et al. [73]. In Cameroon, Bambara groundnut accessions were collected from different locations to contrast their traits for improvement by Ndiang et al. [74] and Sobda et al. [75]. Among the 150 accessions, average days to 50% flowering was found close to 38 days in Malaysia which is lower than the 67.65 days reported by Mohammed [63] out of 101 Ghanaian Bambara landraces. Days to 50% flowering was quite diverse among the Bambara groundnut accessions and the estimated values varied from 25 to 51 days, but the variation of flowering time was reported by Goli et al. [60] ranged from 38 to 68 days for 1384 genotypes. Flowering time for the Bambara groundnut is undefined [76] and plays a role as a vital part of the adaption mechanism of a variety to an environment [73]. The flowering times depends on the various complex proceeding of interaction influenced by genetic and/or environmental element [77] and very related trait for annual farming landraces like *Vigna subterranea* (L.) Verdc [78]. Identical findings were discovered by Massawe et al. [13] ranging from 64 to 76 days in South Africa, while Masindeni [18] reported 43-80 days in Bloemfontein, South Africa. Ouedraogo et al. [10] observed flowering time differs from 32 to 53 days in Burkina Faso. In addition, flowering happened between 36 and 53 days among twenty Bambara groundnut accessions in Pretoria, state of South Africa, observed by Goli et al. [37], also he stated that flowering can be influenced by several environmental factors such as day length, temperature, altitude, and soil conditions as well as genotypic factors. When Bambara groundnut is planted in long-day flowering, it is either delayed or stopped since it is a short-day plant. Early flowering implies early maturity [79], and in our findings, the early flowering accessions S1G108, S1G56, S1G88, S1G75, S1G28, S1G4, S1G13, S1G26, S1G11, S1G29, S1G148, S1G133, S1G49, S1G14, and S1G118 could be selected for early (below 120 days) maturity. Days to maturity was differed significantly (*p* ≤ 0.01) among the accessions and varies from 103 to 163 days, which was more or less similar range reported by Masindeni [18] and Goli et al. [60]. The maturity duration of the Bambara groundnut depends on the cultivar and climatic situation, from 3 to 6 months [79]. Long photoperiods cause delayed maturity of the Bambara groundnut stated by Linneman et al. [80]. In our study, plant height also showed a significant difference (14.23 to 36.9 cm), while all other morphological traits also exhibited variation significantly. Mohammed [63] stated that average values achieved for this trait in his work are supported by this current study. The outcome founded by Ntundu et al. [1] in Tanzania and Goli et al. [37] in South Africa also supported our findings. A strong significant difference (*p* ≤ 0.01) was found between yield and yield contributing traits such as biomass fresh and dry weight, pod and seed number per plant, fresh and dry pod weight, dry seed weight, pod length and width, mature and immature pods per plant, and hundred seed weight showed high genetic variation among these traits. A similar variation in yield contributing traits was also stated by Goli et al. [37] and suggested that these variations due to genotype by environment (G × E) effect on Bambara groundnut yield. In our assessment hundred seed weight, dry pod weight, and total no. of pods varied from 196.21 g to 364.09 g, 88.89 g to 402.9 g, and 38 to 81, respectively. It has been detected that hundred seed weight was considered a vital tool for the judgment of morphophysiological traits related to yield ([1, 10, 13]; [18, 63, 81]). Our calculated yield ranged from 370.38 to 1679 kg/ha, and this finding was supported by Adebisi et al. [62]; his observation was between 146.6 and 2678.6 kg/ha among 52 landraces in Benin; also the study was carried on by [10] in Burkina Faso. Based on our recorded yield, selected 23 elite accessions were identified. These 23 accessions are fitted to cluster II that produced maximum yield and together with 21 accessions of cluster IV created 44 accessions that gave higher yield compared to other accessions evaluated in this study and considered elite accessions. An average 1537.18 kg/ha (29.45%) yield was recorded for 23 accessions of cluster II while the 21 accessions of cluster IV produced 1226.56 kg/ha, which is 23.5% of the total yield. We observed the minimum (5.95%) yield gap between these two groups related to other cluster (I, III, and V). Moreover, cluster II produced 46.51% higher mean yield than the average grand mean yield of 1049.19 kg/ha followed by cluster IV (16.90%). Considering all the parameter studied, a significant relationship was found between the accessions of cluster II and IV in relation to yield and its contributed traits compared to other groups of accessions and this finding were supported by Onwubiko et al. [35]. On the other hand, FAO in 2014 measured the yield of the Bambara groundnut which is lower than the calculated mean yield (1049.19 kg/ha) in our research. A similar observation was noted inW Africa, which was 703.3 kg/ha from the cultivable land of 158,635 hectares [27]. But for the yield of groundnut (*Arachis hypogaea* L.) which was introduced from South America, the calculated yield was 1058.8 kg/ha from a cultivated area of 6,207,414 hectare in West Africa; for this reason, Bambara groundnut was treated as a neglected and underutilized crop due to its low production in West Africa [82].

### 5.3. Correlation (Association) Matrix

The correlations assessment indicated a relationship of some morphological traits with the characters of yield. The association or correlation is an influential tool for the researchers to prefer the traits to be integrated into the genotype selection program [63]. The correlation coefficient is an essential measure of an index in plant breeding; after all, it is the measurement of the magnitude of the correlation between genetic and nongenetic two or more variables. Our findings were supported to those achieved by Mohammed [63] in Cote d'Ivoire, [66] observed among 52 landraces in Benin, and [31] in Cameroon. The accessions with large size seeds fulfill the demand of the farmers as well as consumers and often treated as good commercial qualities [83]. Adebisi et al. [62] declared that for the selection of superior accessions the consideration of correlation values among the variables is a great index of the selection process. Total pod number detected positive and moderate highly significant correlation with yield and positively strong correlation was noted with the number of mature pods, dry seed weight, and hundred seed weight; these results are consistent with the findings of [71], variation correlation [84] studies among yield with its related components. Furthermore, previous researches have reported in the notification by Ntundu et al. [1], Ouedraogo et al. [10], Onwubiko et al. [35], and Goli et al. [37] that the significantly positive correlated traits found in this current research were significantly correlated with seed yield in Bambara groundnut. Consistently, these traits can be directly selected for yield improvement. We observed that there was a positive significant association of plant height and other yield-related traits like the number of total pods, no. of mature pods, fresh pod weight, pod length, seed length, dry seed weight, and hundred seed weight with field yield kg/ha, may be suggested that the selection considering these traits may be useful for yield improvement of Bambara groundnut as well as fodder production.

### 5.4. Genetic Parameter Analysis

#### 5.4.1. Variance and Covariance, Heritability in a Broad Sense, Relative Differences, and Genetic Advances

In past research findings on heritability, it was reported that the selection constructed for certain trait improvement does not only depend on available genetic variation but also the degrees of heritability for such variations [42, 85]. Besides, the valuation of heritability alongside genetic advance contributes a depth advantage over the sole use of heritability [86, 87]. The analysis of the variance elements viz. phenotypic variance and genotypic variance exhibited that phenotypic values were marginally higher than the respected genotypic values for all the traits, are the indication of the trait's expression are influenced by the environment. These findings of our research were supported by previous reporters [88]. Further, the coefficient of genotypic and phenotypic variation results was evaluated based on the statement of the research of [46, 47, 89]. They suggested that the values of the genotypic coefficient of variation (GCV) and phenotypic coefficient of variation (PCV) categorized for low (0% to 10%), for intermediate (10%-20%), and high (≥20%) variation. Based on these criteria, our research results noted that both genotypic coefficients of variation (GCV) and phenotypic coefficient of variation (PCV) were medium to high for most of the traits. Generally, the selection will be fruitful for the development of traits associated with the degree of desirable variation [90]. In this current research, almost all the traits related to yield exposed medium to strong heritability and genetic advance values except the trait's branch number per plant, no. of nodes per stem, and no. of immature pod per plant. So, these traits were significantly remarkable for the selection procedure; however, the traits were controlled by the additive genes with limited response to the environment. This result was supported by Meena et al. [91] and Oladosu et al. [92]. Oppositely, the lower level of genetic advance along with low heritability points out the role of nonadditive genes on these traits, which could be possible to enhance over heterosis breeding [93]. Hence, it is meaningful to prefer those traits with an improved genotypic coefficient of variation (GCV), phenotypic coefficient of variation (PCV), heritability, and genetic advance [94]. Only a powerful selection can be achieved when the effects of additive genes are adequately stronger than the effects of the environment [95]. The greater divergence between the genotypic coefficient of variation (GCV) and phenotypic coefficient of variation (PCV) values is the indication of high effects of the environment to a certain trait whereas the smaller divergence is the indication of strong and significant result of accessions on detectable expression including limited effects of the environment [95].

The estimated result of relative difference (RD) was very high for traits like branch number per plant, no. of nodes per stem, pod width, and no. of immature pod per plant. Bello et al. [96] and Umar et al. [42] confirmed similar findings in their research that the variations were present almost due to the effect of the environment since the improvement of traits cannot be attained by direct selection. Reversely, the characters that had minimum relative differences were due to a genetic effect, indicating that the divergence that exists in these traits can be acquired through direct selection [77].

Estimation of heritability and genetic advance play a vital role in assuming the divergence of phenotypic values which were broadly considered as breeding values. Johnson et al. [49] and Assefa et al. [50] graded the heritability measure as between 0 and 30% for low, 30 and 60% for intermediate, and ≥60% for high. Supposedly, most of the traits considered in this work had high heritability values parallel to high genetic advances. Apparently, in our findings, the traits like biomass fresh (Hb = 98.52%, GA = 82.86%) and dry (Hb = 95.19%, GA = 80.57%) weight, fresh pod weight (Hb = 99.89%, GA = 67.95%), dry pod weight (Hb = 99.75%, *GA* = 62.02%), dry seed weight (Hb = 95.28%, GA = 51.64%), and yield kg/ha (Hb = 99.75%, *GA* = 62.03%) had high heritability alongside with high genetic advance and suggested greater additive effect of genes which provide effective selection for traits improvement directly. This result has an uninterrupted background by the previous research of [85, 90, 96]. The traits with intermediate heritability values also considered as the influence of environmental effects [97]. Onwubiko et al. [98] and Jonah et al. [99] estimate the genetic parameters in Bambara Groundnut with the similar findings of my research output. Besides, the traits with low heritability and genetic advance indicated that the estimated result because of non-additive provably (dominance and or epistasis) genes and/or effects of environment or combined effects of these dual factors. It has been declared by Cornelius [100] that the trait selection with low and moderate heritability values together with low genetic advance may be delayed in traits improvement till their genetic effects get high on over the effect of the environment [101]. Finally, it is evident from the current research that the improvement of yield and other yield contributing traits of *Vigna subterranea* (L.) Verdc. can be obtained through selection by the measurement of heritability and genetic advance.

### 5.5. Cluster Analysis

In the present, investigation of clustering was supported in the past research, observed by Unigwe et al. [43], Gbaguidi et al. [66], Sobda et al. [75], and Bonny et al. [102] in their studied significant variation regarding morphological characteristics distributed in Bambara groundnut, [96] in chili pepper for high yield and CMT values, [83] in cowpea genotypes, and [103, 104] in *Capsicum annuum* L. genotypes. The cluster analysis based on the UPGMA model using numerical traits constructed four distinct groups of Bambara groundnut genotypes in south Africa reported by Unigwe et al. [43], and Atoyebi et al. [105] also constructed dendrogram using statistical analysis software (SAS version 9.3) among 300 accessions of Bambara groundnut.

### 5.6. Estimation of Principal Component Analysis

Typically, the principal component analysis (PCA) is the rejustification tool of cluster analysis. Genetically, identical accessions were clustered into the same group stated by Falconer [50] also genetically dissimilar parents can cover a high degree of heterosis. Johnson [106] noted that principal component analysis intends to determine the total variation that exists in a set of traits which sequentially accounts for the maximum variability in the data. Generally, traits are inter-correlated to varying level and hence all the principal components are not required to summarize the data effectively. The first axes (PC1) elucidate the utmost portion of the total variation in any PCA [107]. Our observation was supported by several types of the research reported by Bello et al. [96], Farhad et al. [108], and Maqbool et al. [109]. Shegro et al. [37] grouped the 20 Bambara groundnut accessions by PCA analysis based on quantitative traits. In our finding's variation percentages of PC1 and PC2 are 34.29% and 11.63% while [110] identified that PC1 and PC2 highly donated to the total variation at 19% and 14%, respectively, in Bambara groundnut. To cluster the genotypes into groups and subgroups, principal component scores were used because first a few principal components controlled all the information of the original variables [111]. Daudo and Olakojo [112] found the similar output during working on maize genotypes, Mustafa et al. [113] observed comparable findings and decided that selection of characters with greater eigenvalues controlled the diversity among the accessions. Mustafa et al. [114] and Jolliffe [115] also gave attention to using accessions based on component traits.

### 5.7. Estimation of Shannon–Weaver Diversity (*H*′ Index)

In our study, the observed diversity index value was more than 4.93 for most of the traits evaluated. This finding supported by Aliyu et al. [60] reported *H*′ index varied from 1.60 to 2.07 for twenty quantitative traits of Bambara lines, Olukolu et al. [116] reported *H*′ index of nineteen qualitative traits of Bambara groundnut varied from 0.1 to 0.15 and twenty-eight numerical traits of 124 accession of Bambara groundnut which showed *H*′ index values between 0.09 and 0.16 across the four African regions. Bonny *et al.* [102] evaluated the diversity in qualitative traits of Bambara groundnut landraces (*Vigna subterranea* L verdc.) in Cốte d'Ivoire of similar findings with me. Nonetheless, the values of *H*′ index for traits appeared statically more or less similar, suggesting a similar genetic diversity. The report from Alvarez et al. [117], Robert et al. [118], and Thomas et al. [119] showed that Bambara groundnut is a self-pollinated crop; therefore, the diversity level of this crop is influenced by farmers' agricultural practices as well as seed management techniques such as recycling, storing, exchanging and newly introducing of species.

## 6. Conclusion

It is noticeable from this current research that the enhancement of yield and other yield-related traits of Bambara groundnut (*Vigna subterranea* L. Verdc) can be obtained through selection by the determination of different genetic parameters analysis. Additionally, the degree of divergence recorded for almost all the agromorphic variables was studied. However, it can be beneficial to the advancement of agromorphic traits of Bambara groundnut by the plant breeders. The current research also resolved strongly to the perfect association between the morphological traits and the field yield. The yield-related traits like no. of the stem, no. of the petiole, no. of mature pods, biomass fresh and dry weight, fresh and dry pod weight, pod weight, dry seed weight, and yield kg/ha recorded high GCV and PCV values were ≥20% with high genetic advance also a low relative difference. It is evident from this present study that the enhancement of yield and other yield-related traits can be attained through effective selection based on estimates of heritability and genetic advance. The result from the principal component and cluster analyses depict that 23 accessions from cluster II and 21 accessions from cluster IV considered high-yielding accessions and can be suggested as large-sized seed associated with high yield potentials. These 44 elite accessions among 150 of Bambara groundnut lines were suggested to grow for further evaluation via conventional breeding alongside with molecular study for confirming and identifying the best 20 accessions.

## Figures and Tables

**Table 1:** The average temperature, average daily sunshine, and average precipitation of the experimental site during the period of the research (2018-2019).

Month	Temperature (°C)			Daily sunshine	Rainfall (mm)/month
	Max	Min	Average	Hours	
September, 2018	31	23	27	7	280
October, 2018	31	23	27	7	280
November, 2018	31	23	27	6	250
December, 2018	31	23	27	6	250
January, 2019	32	22	27	6	238.8
February, 2019	33	23	28	7	260

**Table 2:** The line-up of Bambara groundnut accessions evaluated in this research.

Genetic materials									
Accession name	Code	Accession name	Code	Accession name	Code	Accession name	Code	Accession name	Code
DunP1-18	S1G1	RokP1-18	S1G31	KarP1-18	S1G61	JataP11-18	S1G91	MaibP1-18	S1G121
DunP2-18	S1G2	Maik12-18	S1G32	MaikP6-18	S1G62	MaikP11-18	S1G92	MaikP3-18	S1G122
MaikP14-18	S1G3	CancP13-18	S1G33	KarP3-18	S1G63	GiiwP12-18	S1G93	MaikP9-18	S1G123
DunP4-18	S1G4	BdilaP11-18	S1G34	KarP4-18	S1G64	BdilaP13-18	S1G94	MaibP4-18	S1G124
DunP5-18	S1G5	RokP5-18	S1G35	KarP5-18	S1G65	JataP13-18	S1G95	MaibP5-18	S1G125
BdilaP8-18	S1G6	GiiwP4-18	S1G36	KarP6-18	S1G66	KarP12-18	S1G96	MaikP2-18	S1G126
DunP7-18	S1G7	MaikP1-18	S1G37	KarP7-18	S1G67	DunP11-18	S1G97	MaikP4-18	S1G127
CancP4-18	S1G8	GiiwP5-18	S1G38	KarP8-18	S1G68	KataP7-18	S1G98	MaibP8-18	S1G128
RokP6-18	S1G9	RokP3-18	S1G39	KarP9-18	S1G69	JataP12-18	S1G99	GiiwP13-18	S1G129
DunP10-18	S1G10	GiiwP15-18	S1G40	KarP10-18	S1G70	KarP11-18	S1G100	MaibP10-18	S1G130
RokP2-18	S1G11	DunP12-18	S1G41	MaibP6-18	S1G71	KataP13-18	S1G101	BdilaP1-18	S1G131
MaikP16-18	S1G12	CancP11-18	S1G42	KataP2-18	S1G72	CancP12-18	S1G102	BdilaP2-18	S1G132
RokP8-18	S1G13	ExSokP11-18	S1G43	KataP3-18	S1G73	DunP8-18	S1G103	BdilaP3-18	S1G133
MaibP7-18	S1G14	KataP11-18	S1G44	KataP4-18	S1G74	RokP7-18	S1G104	BdilaP4-18	S1G134
JataP7-18	S1G15	MaikP15-18	S1G45	GiiwP1-18	S1G75	MaikP7-18	S1G105	GiiwP3-18	S1G135
MaikP13-18	S1G16	DunP13-18	S1G46	KataP6-18	S1G76	GiiwP6-18	S1G106	GiiwP10-18	S1G136
CancP1-18	S1G17	GiiwP16-18	S1G47	GiiwP11-18	S1G77	JataP5-18	S1G107	GiiwP9-18	S1G137
CancP2-18	S1G18	GiiwP8-18	S1G48	KataP8-18	S1G78	MaibP3-18	S1G108	DunP6-18	S1G138
RokP10-18	S1G19	RokP11-18	S1G49	KataP9-18	S1G79	MaibP12-18	S1G109	BdilaP9-18	S1G139
MaikP10-18	S1G20	ExSokP12-18	S1G50	KataP10-18	S1G80	BdilaP12-18	S1G110	ExSokP5-18	S1G140
KataP1-18	S1G21	MaibP9-18	S1G51	MaibP13-18	S1G81	GiiwP2-18	S1G111	ExSokP1-18	S1G141
MaikP8-18	S1G22	KataP12-18	S1G52	RokP4-18	S1G82	JataP2-18	S1G112	ExSokP2-18	S1G142
CancP3-18	S1G23	DunP9-18	S1G53	KataP5-18	S1G83	JataP3-18	S1G113	ExSokP3-18	S1G143
BdilaP5-18	S1G24	BdilaP10-18	S1G54	JataP1-18	S1G84	JataP4-18	S1G114	ExSokP4-18	S1G144
CancP5-18	S1G25	KarP13-18	S1G55	DunP3-18	S1G85	RokP9-18	S1G115	ExSokP13-18	S1G145
CancP6-18	S1G26	RokP12-18	S1G56	KarP2-18	S1G86	JataP6-18	S1G116	ExSokP6-18	S1G146
CancP7-18	S1G27	DunP14-18	S1G57	GiiwP7-18	S1G87	MaikP5-18	S1G117	ExSokP7-18	S1G147
CancP8-18	S1G28	MaibP11-18	S1G58	MaibP2-18	S1G88	RokP13-18	S1G118	ExSokP8-18	S1G148
CancP9-18	S1G29	JataP8-18	S1G59	BdilaP7-18	S1G89	JataP9-18	S1G119	ExSokP9-18	S1G149
CancP10-18]	S1G30	GiiwP14-18	S1G60	BdilaP6-18	S1G90	JataP10-18	S1G120	ExSokP10-18	S1G150

Legend: S: selfed; G: genotype/accessions; P: plant; Dun: Duna; Maik: Maika i; Canc: Cancaraki; Rok: Roko; Bidila: Bidilalle; Jata: Jatau; Maib: Maibargo; Kata: Katawa; Giiw: Giiwa; Kar: Karu; Exsok: Ex-sokoto.

**Table 3:** The list of 27 quantitative morphological traits considered according to IPGRI, IITA, and BAMNET [45].

Sl. no.	Name of the traits	Code	Description and measurement type
(1) Phenological traits (3)			
1	Days to emergence	DTE (d)	The number of days from planting to the arrival of 1^st^ typical leaf on the soil surface.
2	Days to fifty % flowering	D50%F (d)	Taken from seed germination to the arrival of 50% flowering(s)
3	Days to maturity	DTM (d)	Days number from sowing to initial time of harvest

(2) Growth and vegetative traits (9)			
4	Plant height	PH (cm)	Data taken from five plants after ten weeks of sowing. Measured from the soil level to the tip of the topmost terminal leaflet
5	Number of branches per stem	NBPS	Data counted at the time of harvest, randomly from five stems of five healthy plants
6	Number of stems per plant	NStPP	Data counted at the time of harvest, randomly from five healthy plants
7	Number of petioles per plant	NPetPP	Data counted 2 weeks later of 1^st^ flowering, randomly from five healthy plants
8	Number of leaves per plant	NLPP	Data counted 2 weeks later of 1^st^ flowering, the average number of 5 plants
9	Number of nodes per stem	NNdPS	Data counted at the time of harvest, randomly from five stems of five healthy plants
10	Internode length	INdLn (cm)	Data measured at the time of ten weeks later of sowing, the average length of 4^th^ internode randomly from five longest stems of five healthy plants
11	Biomass fresh weight per plant	BFWP (g)	Recorded at harvest, randomly average number of 5 fresh plants
12	Biomass dry weight per plant	BDWP (g)	Weight of dried plant, recorded after maintaining the harvested plant dried in sun or oven (55°C for 48 hrs)

(3) Yield traits (15)			
13	Total no. of pod per plant	TNPPP	Data counted at the time of harvest, randomly average values from 5 plants
14	Mature pod number per plant	NMPP	Data counted during harvesting, randomly average values from 5 plants
15	Immature pod number per plant	NImPP	Data counted during harvesting period, randomly average values from 5 plants using an OHAUS Precision Standard Measuring Scale
16	Fresh pod weight	FPW (g)	Recorded at the time of harvest, randomly average values from 5 plants using an OHAUS Precision Standard Measuring Scale
17	Dry pod weight	DPW (g)	Data measured after drying of pods (12% moisture) at 37°C within three weeks of harvest
18	Length of pod	Pod Ln (mm)	Data were taken within 2 months later of harvest, randomly average length from 5 pods. To measure the length, Digital Vernier caliper (cat. no.: 14-648-17, Fisher Brand Traceable, China) was used
19	Width of pod	Pod WD (mm)	Data were taken within 2 months later of harvest, randomly average length from 5 pods. Digital Vernier caliper (cat. no.: 14-648-17, Fisher Brand Traceable, China) was used to measure the length
20	Number of seed per plant	NSdPP	Data counted after dehusking the all pods, randomly average values from 5 plants
21	Dry seed weight per plant	DSWPP (g)	Data measured after drying of seeds (12% moisture) using an OHAUS Precision Standard Measuring Scale within 2 months of harvest
22	Length of seed	Seed Ln (mm)	Data were taken within 2 months later of harvest, randomly average length from 5 seeds. To measure the length, Digital Vernier caliper (cat. no.: 14-648-17, Fisher Brand Traceable, China) was used
23	Width of seed	Seed Wd (mm)	Data were taken within 2 months later of harvest, randomly average length from 5 seeds. Digital Vernier caliper (cat. no.: 14-648-17, Fisher Brand Traceable, China) was used to measure the length
24	Hundred seed weight	HSW (g)	Data measured of 100 dried seeds (12% moisture) using an OHAUS Precision Standard Measuring Scale within two months of harvest
25	Shelling percentage (%):	Shell%	Data measured within two months of harvest; the ratio of dry seed divided by dry pod weight (at 12% moisture content)
26	Harvest index	HI (%)	Measured using the formula of the ratio of Grain yield (kg per ha.)/biological yield (grain+straw)
27	Yield kg per hectare	Yield (kg/ha)	Data weighted of dried pods (at 12% moisture content) per plot, lastly converted the plot yield to a kilogram per hectare (kg/ha).

**Table 4:** List of fourteen qualitative traits considered according to IPGRI, IITA, and BAMNET [45].

Sl. no.	Qualitative traits	Code	Description	Scale
1	Growth habit	(GrH)	Data were taken after 10 weeks of sowing based on the ratio (*P*/*I*) between fourth petiole (*P*) and fourth internode (*I*) length	(1) Accession of bunch type (*P*/*I* ≥ 9) (2) Accession of semi-bunch type (*P*/*I* = 7-9) (3) Accession of Spreading type (*P*/*I* ≤ 7)
2	Stem hairiness	(StH)	Data recorded after harvest	(1) Absent, (2) sparse, (3) dense
3	First stem color	(FSC)	Data recorded after 2 weeks of planting	(1) Green, (2) reddish, (3) striped
4	Terminal leaflet shape	(TLS)	Data were taken after ten weeks of sowing	(1) Oval, (2) round, (3) elliptic, (4) lanceolate
5	Terminal leaflet color	(TLC)	Data were taken after ten weeks of sowing	(1) Green, (2) red, (3) purple
6	Petiole pigmentation	(PetP)	Data were taken after two weeks of sowing	(1) Green, (2) reddish green, (3) without point
7	Shape of pods	(PoS)	Data measured based on the one-seeded pod, in 2 months of harvest	(1) Hook with ending point on the opposite side (2) Round with ending point on the opposite side (3) Hook with ending point on both sides
8	Color of pods	(PoC)	Data were taken in two months of harvest	(1) Brown-yellowish, (2) reddish-brown, (3) brown, (4) black, (5) purple
9	Pods texture	(PoT)	Data were taken in two months of harvest	(1) Smooth with a little groove, (2) much grooved, (3) much folded
10	Seeds shape	(SeS)	Data were taken from seeds of a one-seeded pod in two months of harvest	(1) Round, (2) oval
11	Seeds color	(SeC)	Recorded in two months after harvest	(1) Cream, (2) red, (3) purple cream, (4) black, (5) black cream
12	Eye color	(EyC)	Recorded in two months after harvest	(1) Absent, (2) black
13	Testa pattern	(TeP)	Recorded in two months after harvest	1. Absent of pattern, (2) full line with marbled striped, (3) entirely dotted spot, (4) few rhomboid spots on one side of the hilum, (5) few rhomboid spots on both sides of the hilum, (6) much rhomboid spot on both sides of the hilum
14	Testa color with eye pattern around the hilum.	(Tc+EyP)	Described in a combination of the testa background with eye patterns	(1) Black butterfly-like an eye with cream testa, (2) without eye with black testa, (3) black like butterfly eye with a black strip on creamy background, (4) without eye with black little dots on creamy background, (5) without eye with light brown testa, (6) without eye with dark brown marbled spots, (7) no eye with light brownish-red testa, (8) no eye with brown stripes on a cream background, (9) cream testa without eye

**Table 5:** Categorization of 150 Bambara groundnut accessions into different units by morphotypes.

Different morphounits of Bambara groundnut										
Unit 1 (14 As)	Unit2 (16 As)	Unit 3 (13 As)	Unit 4(13 As)	Unit 5 (13 As)	Unit 6 (13 As)	Unit 7 (13 As)	Unit 8 (13 As)	Unit 9 (16 As)	Unit 10 (13 As)	Unit 11 (13 As)
DunP1-18	MaikP1-18	CancP1-18	RokP1-18	BdilaP1-18	JataP1-18	MaibP1-18	KataP1-18	GiiwP1-18	KarP1-18	ExsokP1-18
DunP2-18	MaikP2-18	CancP2-18	RokP2-18	BdilaP2-18	JataP2-18	MaibP2-18	KataP2-18	GiiwP2-18	KarP2-18	ExsokP2-18
DunP3-18	MaikP3-18	CancP3-18	RokP3-18	BdilaP3-18	JataP3-18	MaibP3-18	KataP3-18	GiiwP3-18	KarP3-18	ExsokP3-18
DunP4-18	MaikP4-18	CancP4-18	RokP4-18	BdilaP4-18	JataP4-18	MaibP4-18	KataP4-18	GiiwP4-18	KarP4-18	ExsokP4-18
DunP5-18	MaikP5-18	CancP5-18	RokP5-18	BdilaP5-18	JataP5-18	MaibP5-18	KataP5-18	GiiwP5-18	KarP5-18	ExsokP5-18
DunP6-18	MaikP6-18	CancP6-18	RokP6-18	BdilaP6-18	JataP6-18	MaibP6-18	KataP6-18	GiiwP6-18	KarP6-18	ExsokP6-18
DunP7-18	MaikP7-18	CancP7-18	RokP7-18	BdilaP7-18	JataP7-18	MaibP7-18	KataP7-18	GiiwP7-18	KarP7-18	ExsokP7-18
DunP8-18	MaikP8-18	CancP8-18	RokP8-18	BdilaP8-18	JataP8-18	MaibP8-18	KataP8-18	GiiwP8-18	KarP8-18	ExsokP8-18
DunP9-18	MaikP9-18	CancP9-18	RokP9-18	BdilaP9-18	JataP9-18	MaibP9-18	KataP9-18	GiiwP9-18	KarP9-18	ExsokP9-18
DunP10-18	MaikP10-18	CancP10-18	RokP10-18	BdilaP10-18	JataP10-18	MaibP10-18	KataP10-18	GiiwP10-18	KarP10-18	ExsokP10-18
DunP11-18	MaikP11-18	CancP11-18	RokP11-18	BdilaP11-18	JataP11-18	MaibP11-18	KataP11-18	GiiwP11-18	KarP11-18	ExsokP11-18
DunP12-18	MaikP12-18	CancP12-18	RokP12-18	BdilaP12-18	JataP12-18	MaibP12-18	KataP12-18	GiiwP12-18	KarP12-18	ExsokP12-18
DunP13-18	MaikP13-18	CancP13-18	RokP13-18	BdilaP13-18	JataP13-18	MaibP13-18	KataP13-18	GiiwP13-18	KarP13-18	ExsokP13-18
DunP14-18	MaikP14-18							GiiwP14-18		
	MaikP15-18							GiiwP15-18		
	MaikP16-18							GiiwP16-18		

Legend: As: accessions number; P: plant; Dun: Duna; Maik: Maika; Canc: Cancaraki; Rok: Roko; Bidila: Bidilalle; Jata: Jatau; Maib: Maibargo; Kata: Katawa; Giiw: Giiwa; Kar: Karu; Exsok: Ex-sokoto.

**Table 6:** Summary of the 27 traits studied significant variation revealed by analysis of variance.

Traits	Rep (df = 2)	Accessions (df = 149)	Mean ± SEm	Max	Min	St. Dev	LSD	CV%
Phenological traits								
DTE (d)	255.61^∗∗^	5.36^∗∗^	8.38 ± 0.08	16	4	1.82	1.22	21.68
D50%F (d)	780.08^∗∗^	66.93^∗∗^	37.51 ± 0.243	51	25	5.16	1.89	13.75
DTM (d)	155.34^∗∗^	249.59^∗∗^	130.89 ± 0.45	163	103	9.52	5.20	7.27

Vegetative traits								
PH (cm)	365.29^∗∗^	28.86^∗∗^	27.14 ± 0.16	36.9	14.23	3.58	2.48	13.17
NBPP	77.96^ns^	89.3^∗∗^	34.56 ± 0.33	58	11	7.15	9.06	20.68
NStPP	260.95^∗∗^	30.89^∗∗^	14.21 ± 0.16	29	6	3.50	1.84	24.65
NPetPP	1280.34^∗∗^	15342.71^∗∗^	293.31 ± 3.37	420	140	71.64	11.76	24.43
NLPP	11523.06^∗∗^	138084.41^∗∗^	879.94 ± 10.13	1260	420	214.93	35.28	24.43
NNdPS	84.88^∗∗^	11.54^∗∗^	12.06 ± 0.11	18	6	2.37	2.32	19.62
INdLn (cm)	42..12^∗∗^	0.81^∗∗^	3.31 ± 0.032	5.51	1.72	0.70	0.36	21.05
BFWP (g)	476.55^ns^	53102.23^∗∗^	327.47 ± 6.29	637.78	120.27	133.42	26.15	40.74
BDWP (g)	12766.16^∗∗^	15647.3^∗∗^	178.66 ± 3.47	371.26	54.9	73.63	25.87	41.21

Yield traits								
TNPPP	519.50^∗∗^	249.37^∗∗^	60.68 ± 0.48	81	38	10.27	8.89	16.92
NMPP	594.30^∗∗^	240.74^∗∗^	48.61 ± 0.47	72	25	9.98	8.17	20.54
NImPP	5.45^ns^	27.16^∗∗^	12.07±0.17	24	5	3.63	4.02	30.08
FPW (g)	3928.16^∗∗^	50712.01^∗∗^	393.9 ± 6.12	642.08	115.5	129.84	6.82	32.96
DPW (g)	5618.9^∗∗^	17298.6^∗∗^	251.8 ± 3.58	402.9	88.89	75.99	6.07	30.18
PodLn (mm)	864.52^∗∗^	75.36^∗∗^	30.93 ± 0.29	53.54	17.96	6.09	5.67	19.69
PodWD (mm)	294.09^∗∗^	13.34^∗∗^	17.12 ± 0.14	29.13	9.74	2.88	3.16	16.83
NSdPP	1238.63^∗∗^	240.74^∗∗^	65.94 ± 0.48	89	43	10.13	8.17	15.36
DSWPP (g)	3069.80^∗∗^	7274.22^∗∗^	190.18 ± 2.36	306	70.5	50.06	17.46	26.32
SeedLn (mm)	198.70^∗∗^	11.68^∗∗^	13.78 ± 0.11	22.48	7.54	2.44	2.15	17.71
SeedWd (mm)	129.01^∗∗^	129.01^∗∗^	11.59 ± 0.92	19.15	6.97	1.95	1.70	16.81
HSW (g)	9339.53^∗∗^	2601.48^∗∗^	270.19 ± 1.56	364.09	196.21	33.16	27.52	12.27
Shell%	35.88^ns^	95.39^∗∗^	76.58 ± 0.30	87.57	52.55	6.48	6.30	8.46
HI	66.98^∗∗^	262.23^∗∗^	58.93 ± 0.45	82.88	38.21	9.50	3.43	16.13
Yld (kg/ha)	97550.34^∗∗^	300323.14^∗∗^	1049.19 ± 14.93	1679	370.38	316.64	25.32	30.18

Legend: Rep: replication; df: degree of freedom; ns: nonsignificant; SEm: standard error of the mean; St. Dev: standard deviation; Max: maximum; Min: minimum; *p* ≤ 0.05: significant (^∗^); *p* ≤ 0.01: highly significant (^∗∗^); CV: coefficient of variation. DTE: days to emergence (d); D50%F: days to 50% flowering (d); DTM: days to maturity (d); PH: plant height (cm); NBPP: number of branches per plant; NStPP: number of stems per plant; NPetPP: number of petioles per plant; NLPP: number of leaves per plant; NNdPS: no. of nodes per stem; INdLn: internode length (cm); BFWP: biomass fresh weight per plant (g); BDWP: biomass dry weight per plant (g); TNPPP: total no. of pods per plant; NMPP: number of mature pods per plant; NImPP: number of immature pods per plant; FPW: fresh pods weight (g); DPW: dry pods weight (g); PodLn: pod length (mm); PodWd: pod width (mm); NSdPP: number of seeds per plant; DSWPP: dry seed weight per plant (g); SeedLn: seed length (mm); SeedWd: seed width (mm); HSW: hundred seed weight (g), Shell%: shelling percent; HI: harvest index (%); and Yld: yield (kg/ha).

**Table 7:** Mean performance of 18 yield contributing traits for the 23 elite accessions out of 150 Bambara groundnut accessions.

Accessions	DTE	D50%F	DTM	PH	NBPP	BFWP	BDWP	TNPPP	FPW	DPW	PodLn	PodWD	SeedLn	SeedWd	HSW	Shell%	HI	Yld
S1G77	7.33	34	132	28.01	34.33	596.1	327.15	74.33	627.34	388.49	38.72	19.79	13.96	11.56	315.8	67.78	54.31	1618.73
S1G92	9.33	32	137	27.27	30.67	621.41	339.33	71.67	636.85	392.47	39.26	21.19	16.45	13.82	315.73	67.14	53.67	1635.29
S1G32	7.33	36	135.67	25.25	35.33	629.18	343.23	71.33	630.51	390.34	37.47	15.07	16.32	14.29	299.23	63.23	53.22	1626.41
S1G137	9.33	36	134.67	30.7	33	614.38	338.34	73	625.01	384.62	36.13	22.49	16.45	13.59	320.52	69.8	53.22	1602.6
S1G2	9.33	35	135.33	23.73	31	619.95	336.34	72	614.23	381.84	39.47	17.81	16.6	13.98	310.37	71.13	53.18	1591.02
S1G17	7	38	135	25.3	31.67	605.51	330.37	70	610.23	374.68	36.48	20.21	15.09	11.9	291.91	64.08	53.16	1561.14
S1G53	8.33	34	124.33	30.81	37.67	533.07	290.71	68.67	587.23	366.53	36.64	14.73	14.59	12.77	290.53	65.89	55.78	1527.23
S1G113	9.33	30	134.33	25.49	36.33	554.16	289.74	74.67	585.56	368.67	35.44	14.91	14.05	12.03	308.69	69.59	56.03	1536.12
S1G63	7.33	30	141.33	27.43	37.67	295.67	156.33	68.67	598.56	371.77	27.02	17.51	12.22	10.32	284.02	64.2	70.48	1549.03
S1G144	13.33	40	146	29.98	36.67	263.27	145.03	73.67	618.21	381.25	34.2	22.59	17.46	14.66	303.66	66.05	72.45	1588.56
S1G62	7.67	36	131	25.73	35	610.54	333.51	75.67	627.23	384.56	39.68	17.84	15.76	13.67	313.62	67.96	53.6	1602.31
S1G70	7	30	137.33	26.34	40.67	271.58	150.25	66	581.93	362.1	40.39	14.09	12.54	10.99	283.01	63.84	70.72	1508.75
S1G150	10.33	37	140	30.34	36.33	319.04	173.57	66.67	581.53	360.31	33.17	19.14	16.79	13.84	297.62	68.15	67.76	1501.3
S1G75	8.33	34	113.67	27	30	220.37	118.43	71.67	617.68	382.88	31.72	17.82	16.09	13.25	309.79	67.32	76.39	1595.32
S1G122	7.33	33	122.67	28.79	33	273.31	143.45	70	623.56	386.68	40.23	18.85	16.92	13.15	300.24	64.08	72.97	1611.2
S1G138	7.67	37	136	34.7	39.67	196.71	108.13	68	568.21	352.19	29.13	18.49	14.92	12.57	286.3	69.3	76.51	1467.46
S1G143	14.33	38	136	33.35	38	193.87	111.69	74.67	587.21	366.14	32.69	17.25	17.15	14.47	329.58	75.83	76.64	1525.58
S1G93	7	34	136	31.94	38.33	306.88	165.59	68.33	638.85	391.89	31.61	17.64	14.64	12.14	277.14	59.03	70.32	1632.87
S1G74	8.33	39	144.33	29.54	35	298.39	162.95	77	602.54	374.07	26.68	15.56	10.93	9.14	310.57	71.73	69.69	1558.65
S1G78	9.33	31	132.67	29.85	36	288.7	151.77	72.33	505.34	317.58	29.6	16.24	16.57	14.29	305.48	78.75	67.67	1323.26
S1G108	7.33	29	106.33	28.54	36.67	298.66	163.06	68	526.9	328.56	29.6	15.06	14.51	11.9	277.91	75.94	66.88	1369.01
S1G107	7.33	35	136.33	26.51	34	616.52	336.53	70	581.9	364.02	39.58	17.05	15.9	12.54	299.28	69.64	51.99	1516.74
S1G34	7	41	137	30.03	32	208.59	110.04	54	498.51	313.61	27.06	14.23	11.84	10.12	240.8	58.31	74.03	1306.72

Legend: DTE: days to emergence (d); D50%F: days to 50% flowering (d); DTM: days to maturity (d); PH: plant height (cm); NBPP: number of branches per plant; BFWP: biomass fresh weight per plant (g); BDWP: biomass dry weight per plant (g); TNPPP: total no. of pods per plant; FPW: fresh pods weight (g); DPW: dry pods weight (g); PodLn: pod length (mm); PodWd: pod width (mm); SeedLn: seed length (mm); SeedWd: seed width (mm); HSW: hundred seed weight (g); Shell%: shelling percent; HI: harvest index (%); and Yld: yield (kg/ha).

**(a):** 

Traits	DTE	D50%F	DTM	PH	NBPP	NStPP	NPetPP	NLPP	NNdPS	INdLn	BFWP	BDWP
DTE	1	0.181^∗∗^	0.130^∗∗^	0.317^∗∗^	-0.01	0.308^∗∗^	0.045	0.045	-0.011	-0.286^∗∗^	-0.084	-0.029
D50%F		1	-0.016	-0.023	-0.116^∗^	0.056	-0.016	-0.016	-0.190^∗∗^	-0.225^∗∗^	-0.08	-0.035
DTM			1	0.035	0.047	0.027	0.002	0.002	0.05	0.095^∗^	0.195^∗∗^	0.192^∗∗^
PH				1	0.021	0.149^∗∗^	0.056	0.056	0.130^∗∗^	0.002	0.003	0.052
NBPP					1	0.113^∗^	0.045	0.045	0.035	0.164^∗∗^	0.150^∗∗^	0.155^∗∗^
NStPP						1	-0.074	-0.074	0.042	-0.196^∗∗^	0.258^∗∗^	0.286^∗∗^
NPetPP							1	1.00^∗∗^	0.00	0.208^∗∗^	0.19^∗∗^	0.186^∗∗^
NLPP								1	0.00	0.208^∗∗^	0.19^∗∗^	0.186^∗∗^
NNdPS									1	-0.007	-0.005	0.018
INdLn										1	0.158^∗∗^	0.098^∗^
BFWP											1	0.981^∗∗^
BDWP												1

**(b):** 

Traits	TNPPP	NMPP	NImPP	FPW	DPW	PodLn	PodWD	NSdPP	DSWPP	SeedLn	SeedWd	HSW	Shell	HI	Yld
DTE	-0.065	-0.041	-0.071	0.001	0.047	0.246^∗∗^	0.054	-0.03	0.091	0.306^∗∗^	0.316^∗∗^	0.158^∗∗^	0.096^∗^	0.056	0.047
D50%F	-0.354^∗∗^	-0.372^∗∗^	0.023	-0.263^∗∗^	-0.226^∗∗^	-0.149^∗∗^	-0.108^∗^	-0.389^∗∗^	-0.210^∗∗^	-0.002	-0.014	-0.265^∗∗^	0.135^∗∗^	-0.142^∗∗^	-0.226^∗∗^
DTM	0.192^∗∗^	0.196^∗∗^	0.004	0.303^∗∗^	0.296^∗∗^	0.217^∗∗^	0.194^∗∗^	0.205^∗∗^	0.310^∗∗^	0.178^∗∗^	0.140^∗∗^	0.181^∗∗^	-0.089	0.043	0.296^∗∗^
PH	0.160^∗∗^	0.145^∗∗^	0.053	0.214^∗∗^	0.245^∗∗^	0.121^∗^	0.011	0.137^∗∗^	0.239^∗∗^	0.355^∗∗^	0.353^∗∗^	0.263^∗∗^	-0.110^∗^	0.121^∗^	0.245^∗∗^
NBPP	0.141^∗∗^	0.169^∗∗^	-0.064	0.191^∗∗^	0.190^∗∗^	0.109^∗^	0.022	0.158^∗∗^	0.216^∗∗^	0.059	0.048	0.148^∗∗^	-0.025	-0.005	0.190^∗∗^
NStPP	0.131^∗∗^	0.214^∗∗^	-0.219^∗∗^	0.205^∗∗^	0.231^∗∗^	0.278^∗∗^	0.061	0.213^∗∗^	0.260^∗∗^	0.289^∗∗^	0.255^∗∗^	0.316^∗∗^	-0.004	-0.087	0.231^∗∗^
NPetPP	-0.067	-0.049	-0.053	0.031	0.033	0.05	-0.013	-0.053	0.036	0.107^∗^	0.120^∗^	-0.062	-0.011	-0.180^∗∗^	0.03355
NLPP	-0.067	-0.049	-0.053	0.031	0.033	0.05	-0.013	-0.053	0.036	0.107^∗^	0.120^∗^	-0.062	-0.011	-0.180^∗∗^	0.033
NNdPS	0.202^∗∗^	0.168^∗∗^	0.109^∗^	0.163^∗∗^	0.172^∗∗^	-0.006	-0.048	0.122^∗∗^	0.156^∗∗^	0.117^∗^	0.098^∗^	0.134^∗∗^	-0.098^∗^	0.134^∗∗^	0.172^∗∗^
INdLn	0.053	0.088	-0.092	0.005	-0.049^∗^	-0.068	0.205^∗∗^	0.099^∗^	-0.042	-0.140^∗∗^	-0.147^∗∗^	-0.113^∗^	0.015	-0.154^∗∗^	-0.049
BFWP	0.281^∗∗^	0.336^∗∗^	-0.130^∗∗^	0.380^∗∗^	0.379^∗∗^	0.246^∗∗^	0.155^∗∗^	0.333^∗∗^	0.367^∗∗^	0.211^∗∗^	0.218^∗∗^	0.330^∗∗^	-0.189^∗∗^	-0.661^∗∗^	0.379^∗∗^
BDWP	0.271^∗∗^	0.326^∗∗^	-0.131^∗∗^	0.375^∗∗^	0.385^∗∗^	0.257^∗∗^	0.120^∗^	0.315^∗∗^	0.379^∗∗^	0.250^∗∗^	0.256^∗∗^	0.350^∗∗^	-0.178^∗∗^	-0.674^∗∗^	0.384^∗∗^
TNPPP	1	0.936^∗∗^	0.253^∗∗^	0.687^∗∗^	0.681^∗∗^	0.300^∗∗^	0.207^∗∗^	0.921^∗∗^	0.702^∗∗^	0.245^∗∗^	0.221^∗∗^	0.880^∗∗^	-0.239^∗∗^	0.273^∗∗^	0.681^∗∗^
NMPP		1	-0.103^∗^	0.672^∗∗^	0.665^∗∗^	0.340^∗∗^	0.262^∗∗^	0.986^∗∗^	0.710^∗∗^	0.252^∗∗^	0.222^∗∗^	0.937^∗∗^	-0.174^∗∗^	0.210^∗∗^	0.665^∗∗^
NImPP			1	0.093^∗^	0.096^∗^	-0.085	-0.136^∗∗^	-0.107^∗^	0.031	0.001	0.013	-0.087	-0.197^∗∗^	0.193^∗∗^	0.096^∗^
FPW				1	0.994^∗∗^	0.374^∗∗^	0.192^∗∗^	0.665^∗∗^	0.937^∗∗^	0.343^∗∗^	0.306^∗∗^	0.661^∗∗^	-0.531^∗∗^	0.399^∗∗^	0.994^∗∗^
DPW					1	0.376^∗∗^	0.159^∗∗^	0.649^∗∗^	0.945^∗∗^	0.378^∗∗^	0.341^∗∗^	0.678^∗∗^	-0.529^∗∗^	0.395^∗∗^	1.000^∗∗^
PodLn						1	0.396^∗∗^	0.376^∗∗^	0.379^∗∗^	0.215^∗∗^	0.186^∗∗^	0.427^∗∗^	-0.143^∗∗^	0.052	0.376^∗∗^
PodWD							1	0.319^∗∗^	0.176^∗∗^	0.07	0.032	0.242^∗∗^	-0.035	0.019	0.159^∗∗^
NSdPP								1	0.697^∗∗^	0.237^∗∗^	0.208^∗∗^	0.939^∗∗^	-0.162^∗∗^	0.209^∗∗^	0.649^∗∗^
DSWPP									1	0.382^∗∗^	0.336^∗∗^	0.730^∗∗^	-0.234^∗∗^	0.375^∗∗^	0.945^∗∗^
SeedLn										1	0.934^∗∗^	0.372^∗∗^	-0.152^∗∗^	0.038	0.378^∗∗^
SeedWd											1	0.345^∗∗^	-0.172^∗∗^	-0.001	0.341^∗∗^
HSW												1	-0.163^∗∗^	0.197^∗∗^	0.678^∗∗^
Shell													1	-0.186^∗∗^	-0.529^∗∗^
HI														1	0.395^∗∗^
Yld															1

Legend: ^∗∗^Correlation is significant at the 0.01 level; ^∗^correlation is significant at the 0.05 level; DTE: days to emergence (d); D50%F: days to 50% flowering (d); DTM: days to maturity (d); PH: plant height (cm); NBPP: number of branches per plant; NStPP: number of stems per plant; NPetPP: number of petioles per plant; NLPP: number of leaves per plant; NNdPS: no. of nodes per stem; INdLn: internode length (cm); BFWP: biomass fresh weight per plant (g); BDWP: biomass dry weight per plant (g); TNPPP: total no. of pods per plant; NMPP: number of mature pod per plant; NImPP: number of immature pods per plant; FPW: fresh pod weight (g); DPW: dry pod weight (g); PodLn: pod length (mm); PodWd: pod width (mm); NSdPP: number of seeds per plant; DSWPP: dry seed weight per plant (g); SeedLn: seed length (mm); SeedWd: seed width (mm); HSW: hundred seed weight (g); Shell%: shelling percent; HI: harvest index (%); and Yld: yield (kg/ha).

**Table 9:** Estimation of variance components, relative difference, heritability, and genetic advance of Bambara groundnut accession.

Traits	Mean	(*σ*_*e*_^2^)	(*σ*_*g*_^2^)	(*σ*_*p*_^2^)	PCV (%)	GCV (%)	RD (%)	(*h*_*b*_^2^) %	GA (%) of Mean
DTE (d)	8.38	0.58	1.59	2.17	17.58	15.06	14.38	73.31	26.55
D50%F(d)	37.51	1.39	21.85	23.24	12.85	12.46	3.03	94.03	24.89
DTM (d)	130.89	10.65	79.65	90.30	7.26	6.82	6.08	88.20	13.19
PH (cm)	27.15	2.39	8.82	11.21	12.33	10.94	11.28	78.71	20.00
NBPP	34.56	31.80	19.17	50.96	20.66	12.67	38.67	37.61	16.00
NStPP	14.22	1.31	9.86	11.17	23.51	22.08	6.05	88.26	42.74
NPetPP	293.31	53.56	5096.4	5149.96	24.47	24.34	0.52	98.96	49.88
NLPP	879.94	482.07	45867.4	46349.47	24.47	24.34	0.52	98.96	49.88
NNdPS	12.06	2.10	3.15	5.25	18.99	14.71	22.55	59.99	23.46
INdLn (cm)	3.32	0.05	0.25	0.30	16.56	15.15	8.50	83.72	28.56
BFWP (g)	327.47	265.00	17612.40	17877.40	40.83	40.53	0.74	98.52	82.86
BDWP (g)	178.66	259.21	5129.40	5388.61	41.09	40.09	2.43	95.19	80.57
TNPPP	60.68	30.62	72.92	103.54	16.77	14.07	16.08	70.43	24.33
NMPP	48.61	25.83	71.64	97.47	20.31	17.41	14.27	73.50	30.75
NImPP	12.07	6.25	6.97	13.22	30.12	21.87	27.39	52.73	32.71
FPW (g)	393.90	18.00	16898.00	16916.00	33.02	33.00	0.05	99.89	67.95
DPW (g)	251.80	14.30	5761.40	5775.70	30.18	30.14	0.12	99.75	62.02
PodLn (mm)	30.93	12.44	20.97	33.41	18.69	14.80	20.77	62.77	24.16
PodWD (mm)	17.12	3.86	3.16	7.02	15.48	10.38	32.93	44.98	14.34
NSdPP	65.94	25.83	71.64	97.47	14.97	12.83	14.27	73.50	22.67
DSWPP (g)	190.19	118.13	2385.40	2503.53	26.31	25.68	2.39	95.28	51.64
SeedLn (mm)	13.78	1.80	3.30	5.10	16.38	13.17	19.58	64.67	21.82
SeedWd (mm)	11.60	1.13	2.11	3.24	15.52	12.53	19.26	65.19	20.84
HSW (g)	270.20	293.30	769.39	1062.70	12.06	10.27	14.91	72.40	17.99
Shell%	76.58	15.37	26.67	42.05	8.47	6.74	20.35	63.43	11.06
HI	58.93	4.56	85.89	90.45	16.14	15.73	2.55	94.96	31.57
Yld (kg/ha)	1049.00	248.24	100025.00	100273.24	30.19	30.15	0.12	99.75	62.03

Legend: *σ*_*e*_^2^: error variance; *σ*_*g*_^2^: genotypic variance; *σ*_*p*_^2^: phenotypic variance; *h*_*b*_^2^: heritability in broad sense; PCV: phenotypic coefficient of variation; GCV: genotypic coefficient of variation; RD: relative difference; GA: genetic advance; DTE: days to emergence (d); D50%F: days to 50% flowering (d); DTM: days to maturity (d); PH: plant height (cm); NBPP: number of branches per plant; NStPP: number of stems per plant; NPetPP: number of petioles per plant; NLPP: number of leaves per plant; NNdPS: no. of nodes per stem; INdLn: internode length (cm); BFWP: biomass fresh weight per plant (g); BDWP: biomass dry weight per plant (g); TNPPP: total no. of pods per plant; NMPP: number of mature pods per plant; NImPP: number of immature pods per plant; FPW: fresh pod weight (g); DPW: dry pod weight (g); PodLn: pod length (mm); PodWd: pod width (mm); NSdPP: number of seeds per plant; DSWPP: dry seed weight per plant (g); SeedLn: seed length (mm); SeedWd: seed width (mm); HSW: hundred seed weight (g); Shell%: shelling percent; HI: harvest index (%); and Yld: yield (kg/ha).

**Table 10:** Relative proportion of average grand yield for five clusters revealed by cluster analysis of Bambara groundnut accessions.

Cluster	Accessions number	Accessions	Average yield (kg/ha)	RPGY (%)
Cluster I	59 (39.33%)	S1G37, S1G76, S1G68, S1G83, S1G35, S1G44, S1G97, S1G112, S1G1,S1G67, S1G72, S1G87, S1G110, S1G95, S1G106, S1G9, S1G24, S1G85, S1G130, S1G16, S1G102, S1G91, S1G123, S1G145, S1G117, S1G7, S1G81, S1G142, S1G135, S1G15, S1G45, S1G6, S1G21, S1G40, S1G48, S1G39, S1G120, S1G60, S1G105, S1G22, S1G31, S1G132, S1G36, S1G126, S1G128, S1G140, S1G61, S1G100, S1G47, S1G98, S1G41, S1G115, S1G96, S1G127, S1G86, S1G125, S1G51, S1G121, S1G136	1077.30 (20.64%)	(+) 2.67

Cluster II	23 (15.33%)	S1G77, S1G92, S1G32, S1G137, S1G2, S1G17, S1G53, S1G113, S1G63, S1G144, S1G62, S1G70, S1G150, S1G75, S1G122, S1G138, S1G143, S1G93, S1G74, S1G78, S1G108, S1G107	1537.19 (29.45%)	(+) 46.51

Cluster III	15 (10%)	S1G49, S1G118, S1G64, 1G109, S1G10, S1G25, S1G88, S1G58, S1G73, S1G56, S1G101, S1G94, S1G116, S1G124, S1G133	670.24 (12.48%)	(-) 36.11

Cluster IV	21 (14%)	S1G84, S1G114, S1G69, S1G12, S1G27, S1G54, S1G8, S1G23, S1G66, S1G111, S1G33, S1G129, S1G42, S1G3, S1G79, S1G99, S1G20, S1G38, S1G18, S1G103, S1G71	1226.56 (23.5%)	(+) 16.90

Cluster V	32 (21%)	S1G50, S1G139, S1G4, S1G26, S1G5, S1G59, S1G90, S1G149, S1G89, S1G104, S1G82, S1G119, S1G11, S1G30, S1G80, S1G146, S1G43, S1G147, S1G29, S1G65, S1G57, S1G141, S1G131, S1G55, S1G52, S1G14, S1G46, S1G19, S1G148, S1G134, S1G13, S1G28	707.82 (13.56%)	(-) 32.53

Note: grand average yield: 1049.19 kg/ha; RPGY: relative proportion of grand average yield (%); ‘(+)': yield higher; ‘(-)': yield lower.

**Table 11:** Principal component analysis and Shannon–Weaver diversity index for quantitative traits of Bambara groundnut.

	PC1	PC2	PC3	PC4	PC5	PC6	PC7	PC8	PC9	H´ index (*H*)	Evenness (*HE*) = *H*/*H*_max_
Eigenvalue	9.26	3.14	2.15	1.88	1.39	1.34	1.10	1.08	0.88		
Proportion of variance (%)	34.29	11.63	7.95	6.95	5.14	4.95	4.08	4.01	3.28		
Cumulative variance (%)	34.29	45.92	53.87	60.81	65.96	70.9	74.98	78.99	82.27		
DTE	0.040	0.043	0.196	0.448	0.026	0.243	-0.018	0.029	0.231	5.00	1.00
D50%F	-0.128	0.057	-0.061	-0.041	0.270	0.428	0.172	0.077	-0.180	5.00	1.00
DTM	0.115	0.057	0.063	0.065	0.319	0.318	-0.208	0.175	0.333	5.01	1.00
PH	0.106	0.001	0.296	0.146	0.041	-0.259	0.173	0.429	-0.233	5.00	1.00
NBPP	0.104	0.094	-0.034	0.071	-0.247	-0.037	0.508	0.145	0.366	5.00	1.00
NStPP	0.112	0.108	-0.208	0.231	-0.035	0.069	0.273	-0.171	0.359	4.99	1.00
NPetPP	0.015	0.329	0.432	-0.130	-0.315	0.140	-0.110	-0.181	-0.032	4.98	0.99
NLPP	0.015	0.329	0.432	-0.130	-0.315	0.140	-0.110	-0.181	-0.032	4.98	0.99
NNdPS	0.094	-0.087	0.108	0.067	-0.065	-0.377	-0.386	0.115	0.508	5.00	1.00
INdLn	0.020	0.274	0.195	0.110	0.014	-0.147	0.180	0.580	-0.111	5.00	1.00
BFWP	0.159	0.393	-0.254	-0.222	0.082	-0.058	-0.044	0.041	0.033	4.93	0.98
BDWP	0.162	0.394	-0.252	-0.213	0.080	-0.060	-0.048	0.052	0.033	4.93	0.98
TNPPP	0.294	-0.115	-0.051	-0.006	-0.080	-0.139	-0.112	-0.019	-0.120	5.00	1.00
NMPP	0.299	-0.045	-0.114	0.117	-0.150	-0.091	-0.050	-0.063	-0.164	4.99	1.00
NImPP	-0.001	-0.216	0.186	-0.364	0.206	-0.150	-0.188	0.128	0.124	4.98	0.99
FPW	0.302	-0.089	0.042	-0.181	0.004	0.151	0.098	0.041	0.020	4.96	0.99
DPW	0.302	-0.088	0.044	-0.180	0.003	0.154	0.099	0.041	0.020	4.96	0.99
PodLn	0.195	0.075	-0.060	0.169	-0.067	0.202	-0.295	0.087	0.115	5.00	1.00
PodWD	0.131	0.093	-0.068	0.308	0.125	0.230	-0.361	0.187	-0.267	5.00	1.00
NSdPP	0.299	-0.045	-0.114	0.117	-0.150	-0.091	-0.050	-0.063	-0.164	5.00	1.00
DSWPP	0.301	-0.068	0.020	-0.082	-0.048	0.141	0.112	0.002	-0.023	4.98	0.99
SeedLn	0.191	0.113	0.229	0.138	0.436	-0.179	0.118	-0.317	-0.029	5.00	1.00
SeedWd	0.178	0.129	0.235	0.119	0.433	-0.221	0.120	-0.336	-0.036	5.00	1.00
HSW	0.300	-0.049	-0.114	0.115	-0.146	-0.089	-0.048	-0.062	-0.163	5.00	1.00
Shell	-0.158	0.088	-0.087	0.357	-0.150	-0.096	-0.013	-0.134	-0.112	5.01	1.00
HI	0.081	-0.465	0.256	0.084	-0.087	0.182	0.103	-0.036	-0.012	5.00	1.00
Yld	0.302	-0.088	0.044	-0.180	0.003	0.154	0.099	0.041	0.020	4.96	0.99

Legend: DTE: days to emergence (d); D50%F: days to 50% flowering (d); DTM: days to maturity (d); PH: plant height (cm); NBPP: number of branches per plant; NStPP: number of stems per plant; NPetPP: number of petioles per plant; NLPP: number of leaves per plant; NNdPS: no. of nodes per stem; INdLn: internode length (cm); BFWP: biomass fresh weight per plant (g); BDWP: biomass dry weight per plant (g); TNPPP: total no. of pods per plant; NMPP: number of mature pods per plant; NImPP: number of immature pods per plant; FPW: fresh pod weight (g); DPW: dry pod weight (g); PodLn: pod length (mm); PodWd: pod width (mm); NSdPP: number of seeds per plant; DSWPP: dry seed weight per plant (g); SeedLn: seed length (mm); SeedWd: seed width (mm); HSW: hundred seed weight (g); Shell%: shelling percent; HI: harvest index (%); and Yld: yield (kg/ha).

## Data Availability

All data are provided in full in the results section of this paper.
